# Pulsed dipolar hyperfine spectroscopy for molecular distance measurements in the angstrom to nanometer scale

**DOI:** 10.1126/sciadv.ady5665

**Published:** 2025-07-25

**Authors:** Lucca Sielaff, Annemarie Kehl, Anakin Aden, Andreas Meyer, Marina Bennati

**Affiliations:** ^1^ Research Group EPR Spectroscopy, Max Planck Institute for Multidisciplinary Sciences, Am Faßberg 11, 37077 Göttingen, Germany.; ^2^ Institute of Physical Chemistry, Georg August University of Göttingen, Tammannstraße 6, 37077 Göttingen, Germany.

## Abstract

Hyperfine spectroscopy is a fundamental method in biophysical and material sciences to detect nuclear spins in vicinity of paramagnetic centers, leading to molecular structural information. Among variants of this experiment, only electron-nuclear double resonance (ENDOR) has been established to detect nuclei at interspin distances up to about 1.7 nanometers using ^19^F labels. This limit is dictated by the ENDOR linewidth of 10 to 30 kilohertz, which appeared insurmountable given dipolar broadening of the detected nucleus with the nuclear spin bath. Herein, we present ENDOR experiments based on nuclear sublevel coherence spectroscopy that push the boundaries of ENDOR sensitivity and resolution by one order of magnitude. In particular, we introduce an experiment, in which the electron-nuclear dipolar interaction can be exquisitely extracted from other nuclear broadening mechanisms, thus enabling to access distance distributions. This methodology paves the way for structural studies using ^19^F ENDOR in biomolecular systems. Moreover, it offers opportunities to access spin dynamics in electron-nuclear coupled spin systems.

## Teaser

Time domain ENDOR pushes the boundary of hyperfine spectroscopy to kilohertz-resolution and micromolar concentrations.

## INTRODUCTION

The hyperfine interaction arising from the magnetic coupling between electron and nuclear spins in atoms and molecules provides fundamental insights into their electronic structure and is widespread to extract structural information in chemistry, biology, physics up to material sciences (*1*). Recently, understanding hyperfine interactions has become crucial also in electron-nuclear polarization transfer experiments, which are aimed at enhancing the sensitivity of nuclear magnetic resonance (NMR) spectroscopy (*2*, *3*) down to single spins (*4*). Among magnetic resonance methods that detect hyperfine couplings (HFCs), electron-nuclear double resonance (ENDOR) spectroscopy has recently raised considerable attention due to its capability of resolving dipolar couplings in the solid state down to tens of kilohertz, which is more than two orders of magnitudes smaller than a typical electron paramagnetic resonance (EPR) linewidth of a few megahertz, the latter dictated by the electronic *T*_2_ relaxation (*5*). Similar as in distance measurements between two electron and two nuclear spins, the dipolar part of the HFC can be directly related to structural information through a point-dipolar approximation for the interspin distance. Because dipolar HFCs reflect through-space interactions, they are applicable to both intramolecular (*6*) and intermolecular (*7*) distance measurements. Although NMR-based nuclear-nuclear distance measurements are not restricted to paramagnetic systems, they suffer from overall low spin sensitivity. In contrast, EPR detection offers higher spin sensitivity due to the polarization of electron spins that have a much larger gyromagnetic ratio ( γe/γH1=660 ) as compared to nuclear spins (*8*).

This development has led to the emerging field of ENDOR-based distance measurements using paramagnetic centers and sensitive nuclei such as fluorine (^19^F) as selective spin labels (*6*, *7*, *9*–*18*). Fluorine nuclei, naturally scarce in biomolecules, can be readily incorporated in biomolecules through diverse labeling strategies (*19*–*21*). Moreover, their high magnetic moment (*22*, *23*), similar to protons, results in strong HFC strengths. The methodology provides a complementary tool in structural biology and has allowed distance determination in large biological systems and in cells.

**Fig. 1.**
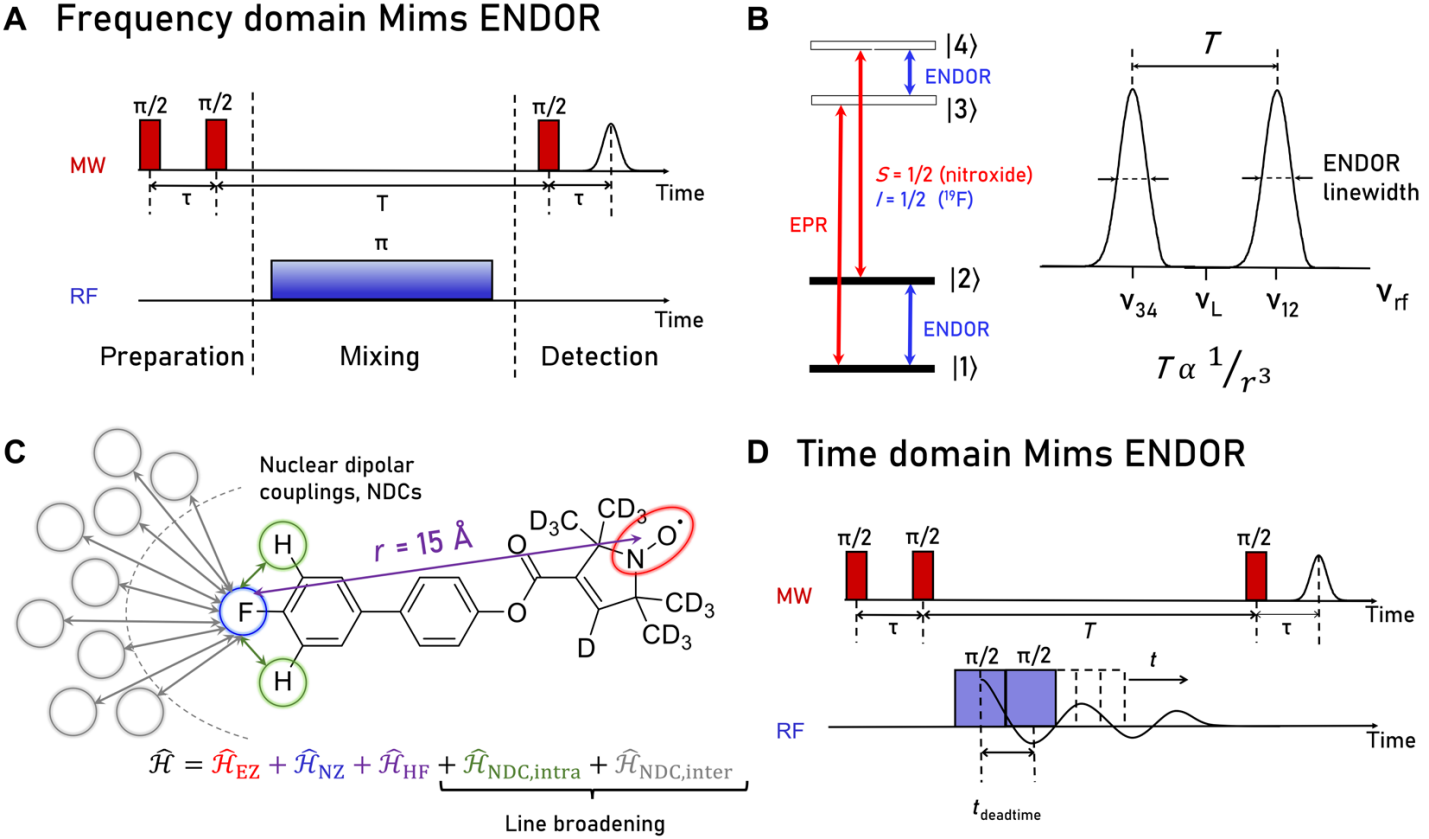
Principle of ENDOR-based molecular distance measurements. (**A**) Pulse scheme of the standard Mims ENDOR experiment to measure small HFCs of <<1 MHz (*6*). The spectrum is recorded in the RF FD, i.e., the RF is changed stepwise through the ENDOR spectrum and the effect on the electron spin echo is recorded. Preparation, mixing, and detection are explained in the main text. (**B**) Left: Simplified energy level scheme of two spins 1/2, showing the allowed EPR ( $∆m_{S}=1$ , 1-3, and 2-4) and NMR/ENDOR transitions ( $∆m_{I}=1$ , 1-2, and 3-4). Right: Corresponding schematic ENDOR spectrum of the two-spin system. The splitting *T* is related to the point-dipole interspin distance *r* (see Eq. 3 further in text). The ENDOR linewidth limits the distance resolution. (**C**) Structure of a model system containing a paramagnetic center *S* = ½ (a nitroxide radical) and a fluorine spin label at a distance of ~15 Å. The general spin Hamiltonian contains two Zeeman terms ( ${\hat{H}}_{\text{EZ}},{\hat{H}}_{\text{NZ}}$ ), describing the interaction with the external magnetic field, the hyperfine interaction ${\hat{H}}_{\text{HF}}$ reporting distance information, and inter- and intramolecular nuclear-nuclear dipolar couplings ${\hat{H}}_{\text{NDC}}$ to the fluorine nucleus. The latter lead to line broadening up to tens of kilohertz and limit resolution. (**D**) Pulse scheme of TD ENDOR (*25*). The RF π pulse in (A) is replaced by a π/2 pulse that creates nuclear sublevel coherences [1-2 or 3-4 in (B)]. These can be read out in electron spin polarization by a second π/2 pulse, stepped through the nuclear FID. The initial part of the oscillation is hidden in the RF pulse length, causing a dead time.

Thus far, an accessible distance range up to 1.5 to 1.7 nm has been established, which depends only slightly on the spin label properties, using the so-called Mims ENDOR experiment (Fig. 1A). The EPR pulse sequence consists of a stimulated echo, with a preparation block of two nonselective microwave (MW) pulses separated by an evolution period τ (*5*). This generates a magnetization grating across the EPR line, which is used to transfer polarization on a target nucleus by a π radio frequency (RF) pulse, on resonance with a hyperfine transition (called mixing block). This polarization transfer causes a depolarization of the electron spin echo, giving rise to the ENDOR effect (detection). The hyperfine spectrum, illustrated schematically for an electron spin coupled to ^19^F in Fig. 1B, is probed by varying stepwise the frequency of the RF π pulse around the Larmor frequency of ^19^F, called frequency domain (FD) acquisition. In this experiment, the full magnetic interactions of the ^19^F spin, illustrated in Fig. 1C, including its chemical shift anisotropy (CSA) and nuclear dipolar couplings (NDCs), contribute to the spectrum. The bandwidth of the RF pulse as well as CSA and NDCs lead to broadening of the hyperfine dipolar spectrum (*24*, *25*). In particular, NDCs to nearby and bulk ^1^H lead to a resolution limit of 10 to 30 kHz.

An alternative approach to measure the Mims ENDOR spectrum is in the RF time domain (TD), as initially proposed by Mehring and colleagues (*26*) and described by Gemperle and Schweiger using product operator formalism (*27*). The main idea is to excite the whole hyperfine spectrum with one single π/2 RF pulse, on resonance with the observed nucleus. This creates nuclear sublevel coherences that evolve during a time interval *t* and can be read back in electron depolarization through a second RF π/2 pulse (Fig. 1D). The ENDOR spectrum is obtained by Fourier transform of the detected nuclear coherences [free induction decay (FID)], taking advantage of higher sensitivity due to multiplex excitation. With this, narrower ENDOR lines can be observed as power broadening is suppressed (*24*, *28*, *29*). The challenge of TD ENDOR is to achieve sufficient bandwidth of the RF π/2 pulse because RF circuits in ENDOR are nonresonant and thus broadband. Because of this limitation [~200 kHz on a commercial Q-band instrument (see Materials and Methods)], TD ENDOR has been rarely used in the literature. The advent of ^19^F ENDOR distance measurements, where very small hyperfine dipolar couplings (<<1 MHz) are detected and thus only small excitation bandwidths are required, offers new opportunities to revisit the TD-ENDOR approach.

Here, we demonstrate unique capabilities of TD ENDOR–based sequences in ^19^F distance measurements. In a first step, we illustrate that a dead-time-free (DTF) detection of the ^19^F spin echo is feasible and allows us to circumvent a more difficult FID reconstruction (see Fig. 1D). We show that the multiplex advantage of Fourier transform permits to record ENDOR spectra of samples at low micromolar concentrations, effectively enhancing the ENDOR sensitivity of about one order of magnitude versus standard FD ENDOR. In the second and main step, we introduce a TD experiment that disentangles the hyperfine interaction from other undesired nuclear spin interactions. This experiment is inspired by the four-pulse double electron electron resonance (DEER) sequence (*30*) between two electron spins, which we now adapt to the case of an electron and a nuclear spin. Using a series of rationally designed model systems, we demonstrate that the method pushes ENDOR resolution by one order of magnitude. Last, we show that new distance distributions become accessible from ENDOR and validate them against distributions derived from density functional theory (DFT)–predicted structures.

## RESULTS

### Model compounds

**Fig. 2.**
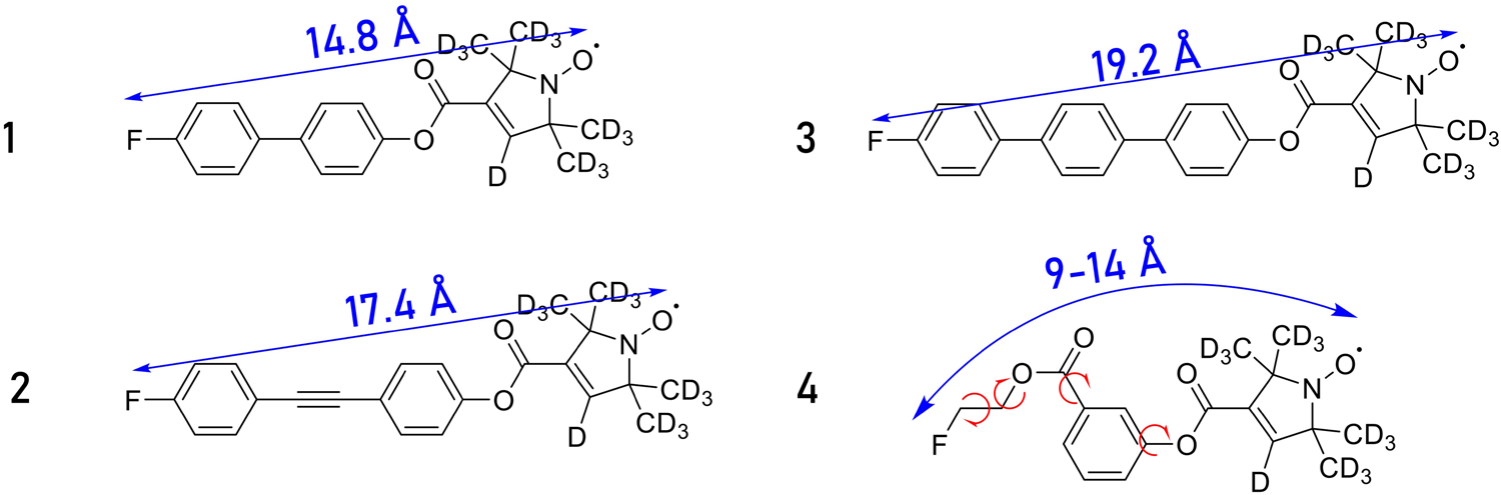
Nitroxide-fluorine model compounds. Chemical structures of nitroxide-fluorine model compounds. Blue arrows indicate the DFT-predicted distances between the radical spin center, localized on the nitrogen-oxygen moiety of the nitroxide, and the ^19^F nucleus. Red arrows indicate flexible bonds that influence the distance distribution of **4**.

To demonstrate our concepts, we used four rationally designed nitroxide-fluorine model compounds (Fig. 2). We synthesized the compounds based on our experience with fluoroaryl-substituted nitroxide model systems (*6*, *24*, *25*). Details of the synthesis are reported in the Supplementary Materials. The ^19^F-electron distances were extracted from DFT calculations (tables S1 to S4) and range from 9 to 20 Å. Model compounds **1 to 3** consist of rigid molecular backbones. As previously shown, rotation around the pyrroline-*N*-oxide moiety leads to conformers that are higher in energy and are therefore only marginally populated (*6*). This leads to narrow distance distributions, making them ideal molecular rulers. In contrast, model compound **4** represents a case of a flexible spin label, which leads to a more complex distance distribution, as discussed later in the text. Because we performed ENDOR experiments at a magnetic field of 1.2 T (34-GHz EPR Larmor frequency), the effect of overlapping ^1^H and ^19^F resonances was circumvented by using deuterated nitroxide labels at the pyrroline-*N*-oxyl moieties. All samples were prepared in solutions of deuterated dimethyl sulfoxide-*d*_6_ (DMSO-*d*_6_)/glycerol-*d*_8_ (2:3) to achieve optimal electron relaxation properties.

### Sensitivity enhancement using DTF-TD ENDOR

**Fig. 3.**
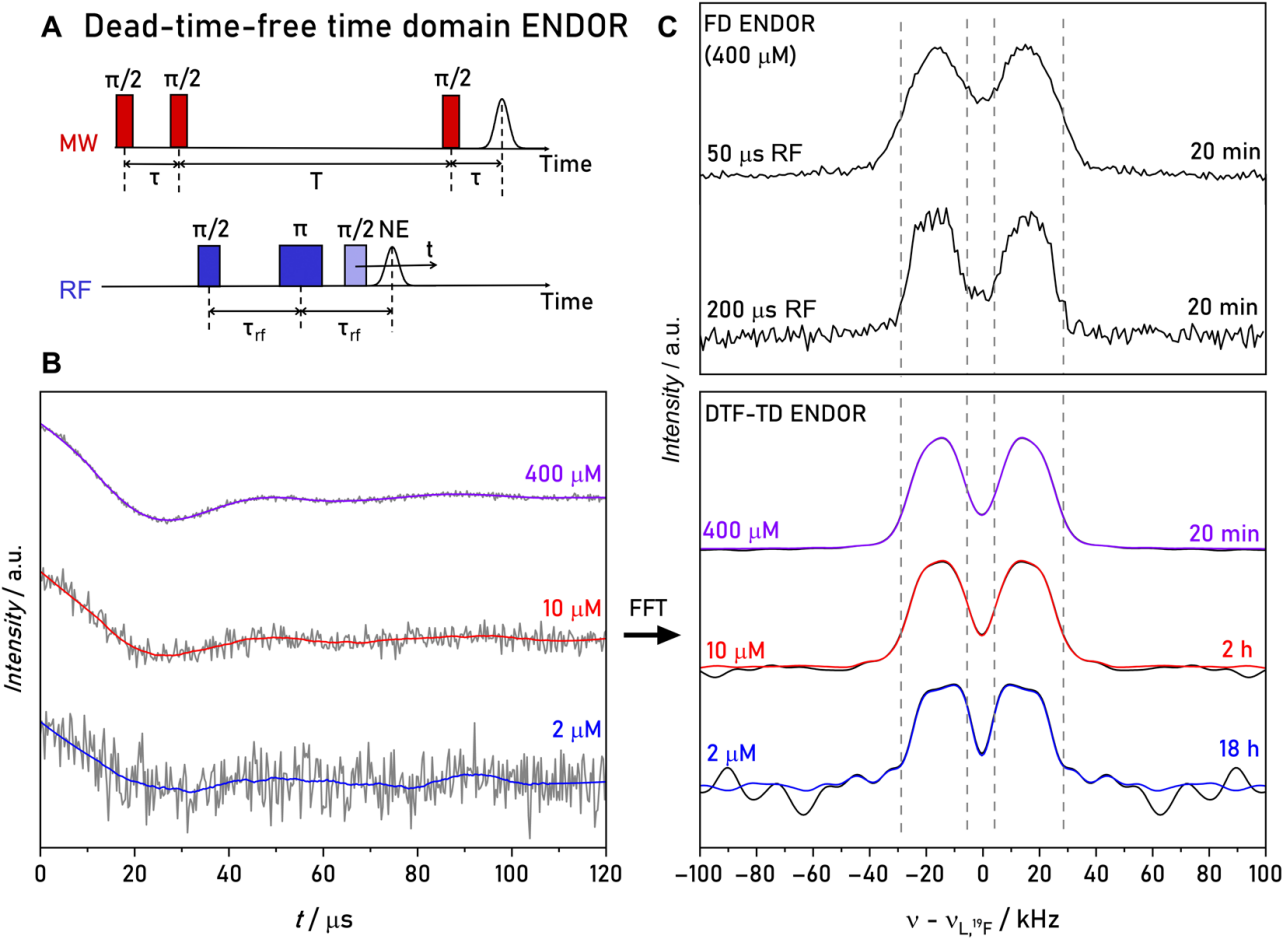
34-GHz, ^19^F DTF, TD ENDOR of model compound 1. (**A**) Schematic pulse sequence of the experiment. An additional π pulse is inserted in the RF block to create a nuclear spin echo (NE). (**B**) TD traces for different sample concentrations recorded at 50 K recorded with 12-ns MW π/2 pulses, 6-μs RF π/2 pulses, τ = 3 μs, τ_RF_ = 8 μs, 8-ms (400 μM) or 6-ms (10 and 2 μM) SRT, and 10 SPP. The last RF pulse was stepped in 240-ns (400 μM) or 300-ns (10 and 2 μM) steps. Gray traces show the raw data. Colored traces are smoothed with a Savitzky-Golay filter (window: 80 points). a.u., arbitrary units. (**C**) Fourier transform of the time traces and comparison of DTF-TD ENDOR with FD ENDOR at 400 μM concentration. FD ENDOR spectra were recorded with 50- and 200-μs RF π pulses, τ = 3 μs, 8-ms SRT, and 1 SPP. Before Fourier transform, all time traces were multiplied by a hamming window and zero filled to 10 times the length of the time trace. Measurements times in minutes (min) or hours (h) are given in the legends. The total number of scans is given in table S5.

In a first step, we examined the performance of TD ENDOR with compound **1**. Starting from the sequence in Fig. 1D, we inserted an additional refocusing RF π pulse to create a nuclear coherence echo and stepped the readout RF π/2 pulse through the time interval, in which the echo was expected. The sequence is shown in Fig. 3A. During the whole RF block (mixing time), the electron spin is stored in longitudinal magnetization, and at a temperature of 50 K relaxes on the millisecond timescale (fig. S1). Therefore, the length of the RF block could be set to hundreds of microseconds. A phase cycle on the three RF pulses allowed us to extract only changes in electron spin echo that were generated by the RF pulses (Materials and Methods). The 34-GHz/1.2-T time traces of the ^19^F nuclear echo of model compound **1**, recorded with the sequence in Fig. 3A, and their Fourier transforms are displayed in Fig. 3 (B and C). The length of the RF π/2 pulse amounted to 4 to 6 μs (fig. S2), corresponding to a bandwidth of ~200 kHz, sufficient to excite a full dipolar spectrum. The zero point of the trace is set to the echo maximum; thus, the experiment is DTF. It was possible to record nuclear coherence evolution up to almost complete decay, which required a time window of 120 μs. One might expect that nuclear coherences decay with *T*_2n_, which is on the order of milliseconds (*24*). However, the anisotropy of the HFCs combined with other nuclear spin interactions leads to a faster decay of the nuclear echo, as discussed in more detail in the next section.

We performed DTF-TD ENDOR experiments of compound **1** with different sample concentrations at the *g*_y_ observer position in the EPR spectrum (fig. S3) and compared with a standard FD ENDOR spectrum (Fig. 3C). Experimental parameters were optimized separately for each method to ensure optimal performance. To compare ENDOR line shapes of both experiments, we minimized power broadening in FD ENDOR experiments using an RF pulse length of 200 μs (*24*). Additional details on the experimental parameters are provided in Materials and Methods.

It is evident that the DTF-TD ENDOR spectra display a similar line shape as FD ENDOR with long RF pulses, albeit with a much higher sensitivity. Although the use of shorter RF pulses increases the sensitivity of FD ENDOR, it leads to power broadening, reducing the resolution of the experiment. Despite the shorter RF pulses, FD ENDOR still cannot achieve the sensitivity of DTF-TD ENDOR (table S6). Notably, the increased sensitivity enables ENDOR measurements at sample concentrations as low as ~2 μM. The concentration of the sample was validated based on a calibration of its EPR signal intensity (fig. S4). We attribute the slight line shape differences in the 2 μM sample to the lower signal-to-noise (SNR). However, the splitting in the spectrum is still clearly resolved. Over all model compounds, DTF-TD ENDOR achieves up to one order of magnitude improved SNR ratio relative to FD ENDOR at comparable resolution and under identical acquisition times (table S6).

### From TD ENDOR to pulsed dipolar hyperfine spectroscopy

**Fig. 4.**
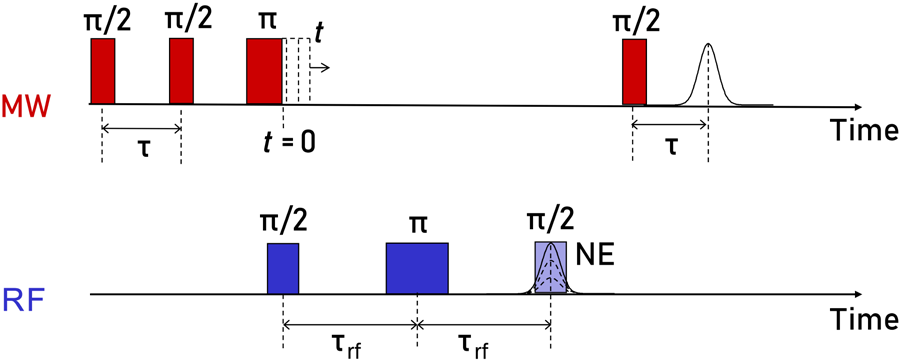
Principle of PDHS. Schematic PDHS pulse sequence and respective time delays. During the DTF-TD experiment, an additional MW pulse is inserted during the RF block and it is stepped through the nuclear coherence evolution τ_RF_. The RF readout pulse is kept fixed, coincident with the maximum of the nuclear echo. Last, the intensity of the stimulated echo is recorded as a function of the incremented time *t* of the additional MW pulse.

The ability to generate and detect nuclear spin coherences using TD ENDOR offers opportunities for manipulating hyperfine interactions. The DEER (*30*) and Spin Echo Double Resonance (SEDOR) (*31*) experiments are two examples of how the transfer of electron and nuclear coherences, respectively, can be used to extract dipolar time evolutions between two spins, refocusing all other spin interactions. Following this idea, we propose the experiment illustrated in Fig. 4 that we call pulsed dipolar hyperfine spectroscopy (PDHS).

We take advantage of the refocused ^19^F nuclear spin echo, shown in Fig. 3, to introduce a dipolar evolution time using an additional π MW pulse on the electron spin. We hypothesize that nuclear spin interactions such as NDCs and CSA can be refocused at the time point of the echo and only the HFC experiences a phase shift through this pulse. We note that, in contrast to the DEER and SEDOR scheme, where the π pulse is selective on the partner spin and otherwise not involved in the sequence, here the additional π pulse is on the observed spin. Without the RF block, the sequence is reminiscent to a HYSCORE experiments; thus, several coherence transfer pathways are expected.

To get a first insight in the sequence, we consider a reduced spin system ( S=1/2 , I=1/2 ) in the high-field approximation with the doubly rotating frame Hamiltonian

$$\hat{H}=\Delta \omega_{S}{\hat{S}}_{z}+\Delta \omega_{I}{\hat{I}}_{z}+A_{\mathrm{zz}}{\hat{S}}_{z}{\hat{I}}_{z}$$ (1)

where $\Delta \omega_{S}$ and $\Delta \omega_{I}$ are the resonance offsets of the electron and nucleus, respectively, and $A_{\mathrm{zz}}$ is the *zz* component of the HFC tensor. The latter generally consists of an isotropic and dipolar contribution, written in tensor form $A=a_{\text{iso}}\cdot 1+T$ (*32*, *33*). In the following we abbreviate $A_{\mathrm{zz}}$ as A . The hyperfine interaction leads to nuclear transition frequencies $\omega_{I}^{\pm }=\Delta \omega_{I}\pm A/2$ . Similar to DTF-TD ENDOR, the first RF π/2 pulse of the PDHS sequence generates nuclear coherences that evolve with $\omega_{I}^{-}$ and $\omega_{I}^{+}$ during the time interval $\tau_{\mathrm{RF}}$ . The following RF π pulse causes a refocusing of these coherences to a nuclear spin echo after a second period $\tau_{\mathrm{RF}}$ . We expect that the additional MW π pulse induces a nuclear coherence transfer, causing spins that formerly evolved with the frequency $\omega_{I}^{+}$ to evolve with $\omega_{I}^{-}$ and vice versa (*30*). Using product operator formalism on the whole Mims sequence (Supplementary Materials and fig. S5), we calculated the stimulated echo intensity as a function of the position *t* of the MW π pulse

$$I\left(t\right)=\frac{1}{2}{\text{sin}}^{2}\left(\frac{A}{2}\tau \right)\cdot \text{cos}\left(\mathrm{At}\right)$$ (2)

Equation 2 shows that the electron spin echo is modulated with a cosine term as a function of the HFC A and the delay time t , which is stepped during the experiment. Furthermore, PDHS, like Mims FD ENDOR, generates blind spots in the spectrum, depending on the time interval τ . It is important to note that the nuclear spin echo is modulated with twice the frequency that we would observe in a (DTF) TD ENDOR spectrum. This is because the conventional ENDOR spectrum shows resonances at frequencies $\omega =\omega_{n}\pm A/2$ , whereas the pulse dipolar spectrum directly reports A , which offers an additional resolution advantage.

### Resolution enhancement with PDHS

**Fig. 5.**
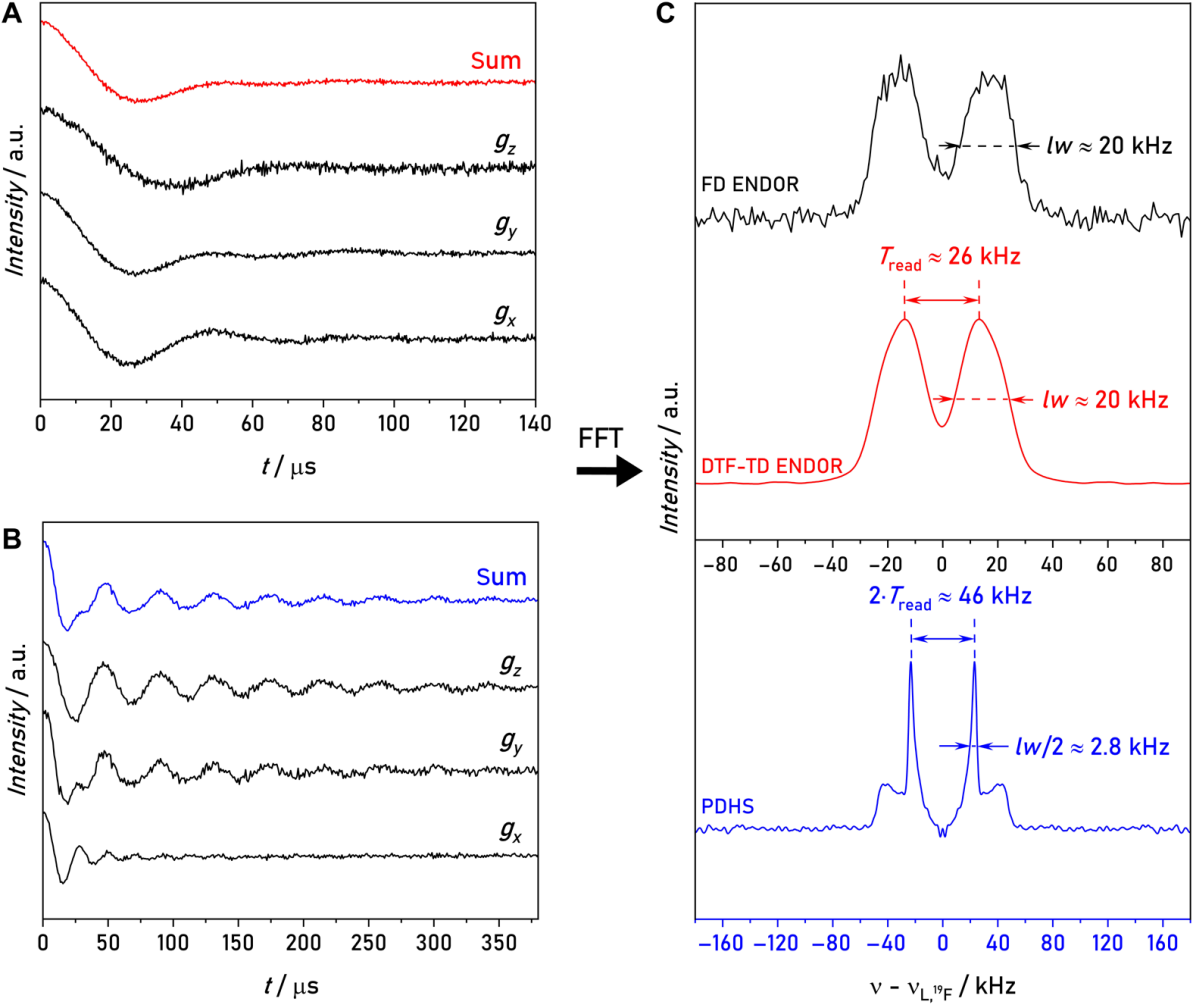
PDHS experiments of model compound 1. (**A**) Comparison of DTF-TD traces of three observer positions (*B*_0_ || *g_x_*, *g_y_*, *g_z_*) in the EPR line and sum time trace. (**B**) Comparison of PDHS traces of three observer positions (*B*_0_ || *g_x_*, *g_y_*, *g_z_*) in the EPR line and sum time trace. All traces were recorded with 12-ns MW π/2 pulses, 6-μs RF π/2 pulses, τ = 3 μs, 8-ms SRT, and 10 SPP. For all DTF-TD traces, a τ_RF_ of 8 μs was used and the last RF pulse was stepped in 240-ns steps until a delay of 120 μs. For all PDHS traces, a τ_RF_ of 426 μs was used and the MW π pulse was stepped in 960-ns steps until a delay of 380 μs. (**C**) Sum spectra of three observer positions acquired with FD ENDOR and Fourier transform of DTF-TD ENDOR and PDHS sum time traces. FD ENDOR spectra were recorded with 200-μs RF π pulses, τ = 3 μs, 8-ms SRT, and 1 SPP. Before Fourier transform, both sum time traces were multiplied by a hamming window and zero filled to 10 times the length of the time trace. FFT, fast Fourier transform.

To verify this concept, we performed PDHS experiments with compound **1**. We inserted the nonselective MW π pulse in the DTF sequence in front of the first RF pulse and noticed that an electron spin echo modulation was observable as soon as this pulse was coincident with the center of the first RF pulse (fig. S6). The acquisition of background signals was attenuated by using RF phase cycling, which removes all signal contributions caused solely by the MW channel (Materials and Methods). To average orientation selection effects, we summed up the time traces recorded at three resonance positions in the EPR line, scaled with respect to their EPR echo signal intensity. In Fig. 5 (left panel), we compared the measured PDHS time traces with respective DTF time traces. It is evident that the PDHS traces show strong modulations and decay much slower than the DTF traces. This different damping must reflect the different interactions involved in the decay. After Fourier transform, the PDHS sum trace reproduces a dipolar Pake pattern in contrast to DTF-TD ENDOR and standard FD ENDOR (Fig. 5, right panel). We note that the observed line shape is a distorted Pake pattern as it is multiplied with the blind spot function in Eq. 2. This multiplication leads to an attenuation toward zero frequencies in the center and enhanced intensity toward the parallel component of the Pake pattern (fig. S7). In the following, we will refer to this line shape as Pake pattern.

In particular, we note that we can resolve the parallel components *T*_||_ of the dipolar tensor, which usually contribute to the initial fast decay of the time trace, as know from four-pulse DEER (*34*). Although the MW π pulse of the PDHS sequence acts on the primary nuclear echo, similarly as in a three-pulse DEER experiment, PDHS behaves effectively DTF like a four-pulse DEER experiment.

When comparing the line shapes of the hyperfine spectra from the three methods (Fig. 5, right panel), DTF-TD ENDOR and FD ENDOR exhibit similar line broadening of ~20 kHz. In PDHS, the full width at half maximum (FWHM) of the main peak is reduced to 5.6 kHz. Given that in PDHS we observe doubling of the Pake frequencies, this width would correspond to 2.8 kHz in the FD spectrum, or a sevenfold improvement in resolution (see Fig. 5).

The higher resolution of the PDHS spectra allows for a more detailed comparison of the extracted tensor values and distances. Assuming a localized electron-spin density, an interspin distance between a nitroxide spin label and ^19^F can be estimated using the point-dipole model (*6*)

$$T_{⊥}=\frac{\mu_{0}}{4\pi h}\left(g_{e}g_{n}\mu_{B}\mu_{N}/r^{3}\right)=C/r^{3}$$ (3)

where $T_{⊥}$ is the perpendicular component of the dipolar hyperfine tensor, $\mu_{0}$ is the vacuum permeability, *h* is the Planck’s constant, and $C=\mu_{0}g_{e}g_{n}\mu_{B}\mu_{N}/4\pi h=74.52\;\text{MHz}\cdot {Å}^{3}$ is calculated from the bohr magneton, the *g* value of the nitroxide ( $g_{\mathrm{iso}}\approx 2.005$ ), and the nuclear magneton and *g* value of ^19^F (*6*). For FD and DTF-TD ENDOR, the splitting *T*_read_ of 26 ± 2 kHz corresponds to an interspin distance of 14.2 ± 0.4 Å. The enhanced resolution of PDHS allows a more accurate determination of $T_{⊥}$ = 23.0 ± 0.3 kHz and $T_{∥}$ = 45.5±0.3kHz corresponding to an interspin distance of 14.8 ± 0.1 Å. This value is in better agreement with the DFT calculations of model compound **1**, which predict a distance of 14.8 Å (see Table 1).

**Table 1.** PDHS distance distributions. Comparison of distance distributions extracted from the PDHS time traces and DFT-predicted distances. Errors were extracted from fitting based on the 95% confidence interval.

Sample	*r*_read_ / Å	〈r〉 / Å	σ / Å	FWHM / Å	*r*_DFT_ / Å
**1**	14.8 ± 0.1	14.65 ± 0.02	0.18 ± 0.02	0.42 ± 0.05	14.8
**2**	17.5 ± 0.2	17.30 ± 0.03	0.36 ± 0.04	0.85 ± 0.10	17.4
**3**	19.5 ± 0.2	18.87 ± 0.10	0.44 ± 0.10	1.04 ± 0.25	19.2

We note that all experiments were recorded with a sample of 400 μM to record full PDHS traces with no compromise in sensitivity. A full trace of the same sample at 100 μM concentration is displayed in fig. S8, and we still achieve an SNR of ≥ 15. As it is well known from four-pulse DEER spectroscopy (*34*), the SNR can be substantially improved by recording shorter time traces, with only a few modulation periods. This should allow us to access much lower concentrations also in PDHS. A systematic study will be object of future work.

### Spin dynamics simulations

The analysis of PDHS with product operator formalism was based on a strongly simplified spin system and does not explore the effects of additional nuclear spin interactions. A key observation in Fig. 5 is that PDHS can remove nuclear dipolar broadening. To verify the fidelity of PDHS in removing NDCs, we performed numerical spin dynamics simulations using software developed in-house (*24*, *33*) and an extended spin system containing three nuclear spins, the ^19^F and the two ^1^H in closest vicinity (Fig. 1C). The separate treatment of the nitroxide spin part with its *g* and ^14^N hyperfine tensor as well as the orientation selection was reported previously (*24*) and is explained in Materials and Methods. The following ENDOR spin Hamiltonian was used in the simulations

H^ENDOR=H^EZ+H^NZ+H^HF+H^NDC,intra=μBgeB0S^z−μNgnB0(1−σ^zz)I^z(F19)+hS^zAzz(F19)I^z(F19)+∑i=12hDzz,i(Hi1)I^z(F19)I^z,i(Hi1) (4)

The individual terms were described in Fig. 1. On the basis of the DFT-optimized structure of model compound **1** (fig. S9), we expect a coupling strength of 6.5 kHz for each of the two neighboring protons to the fluorine nucleus. Because of its negligible influence at 1.2 T (*24*), the CSA was neglected for the calculations. In addition, we used the experimental pulse lengths and time delays of DTF-TD ENDOR and PDHS experiments as input for the simulations. *T*_2e_ = 3 μs and *T*_2n_ = 3 ms values were considered as phenomenological relaxation rates (*24*).

**Fig. 6.**
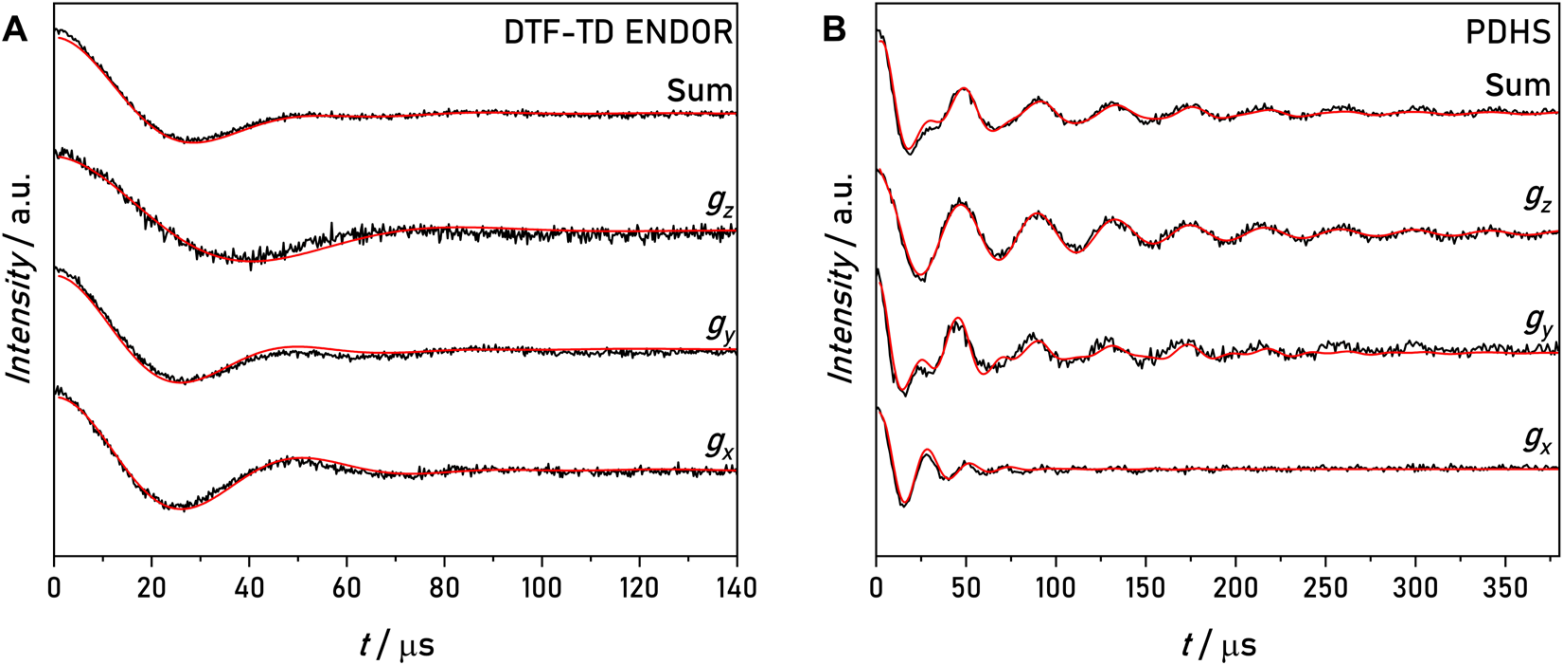
Spin dynamics simulations of model compound 1. Simulations are shown as red solid lines. (**A**) Experimental and simulated DTF-TD ENDOR traces of three observer positions and sum time trace. (**B**) Experimental and simulated PDHS traces of three observer positions and sum time trace. HFC and NDC coupling constants used as input were extracted from DFT-optimized structures of model compound **1**. As simulation parameters, the experimental pulse length and time delays of the respective experiments were used, as well as $T_{∥}=47.4\;\text{kHz}$ with Euler angles [α,β,γ]=[161,1,0]° , two NDCs with $D_{∥}=6.5\;\text{kHz}$ with Euler angles [α,β,γ]=[−17,143,0]° and [−48,34]° , respectively, g=[2.00875,2.00610,2.00211] , A(N14)=[15,11,95.8] MHz, a quadrupole coupling of P=[1.2,0.54,−1.7] MHz, both with all Euler angles equal to 0 [parameters in accordance with (*23*)], *T*_2e_ = 3 μs and *T*_2n_ = 3 ms.

Each calculated time trace was multiplied with an exponential function to achieve the observed oscillation decay  $\begin{array}{c} \left[f\left(t\right)=\text{exp}\left(\;-t/T_{\text{decay}}\;\right),\;\right. \end{array}$ $\begin{array}{c} \left.T_{\text{decay},\text{DTF}-\text{TD}\;\text{ENDOR}}\approx 80\;\mu s,T_{\text{decay},\text{PDHS}}\approx 200\;\mu s\right] \end{array}$. We assume that this decay accounts for the distance distribution not considered in the calculation. The obtained simulations are shown together with the experimental data in Fig. 6. The simulated time traces of DTF-TD ENDOR and PDHS reproduce the effect of the two ^1^H NDC on the respective experiments. Although the two ^1^H-^19^F NDCs lead to the observed line broadening in DTF-TD ENDOR, they are not visible in the PDHS time trace. A further comparison of simulations with and without NDC is shown in fig. S10. This calculation shows that the PDHS traces with or without NDCs are indistinguishable. The results confirm the hypothesis that the PDHS experiment suppresses the NDCs.

### Accessing distance distributions from PDHS

**Fig. 7.**
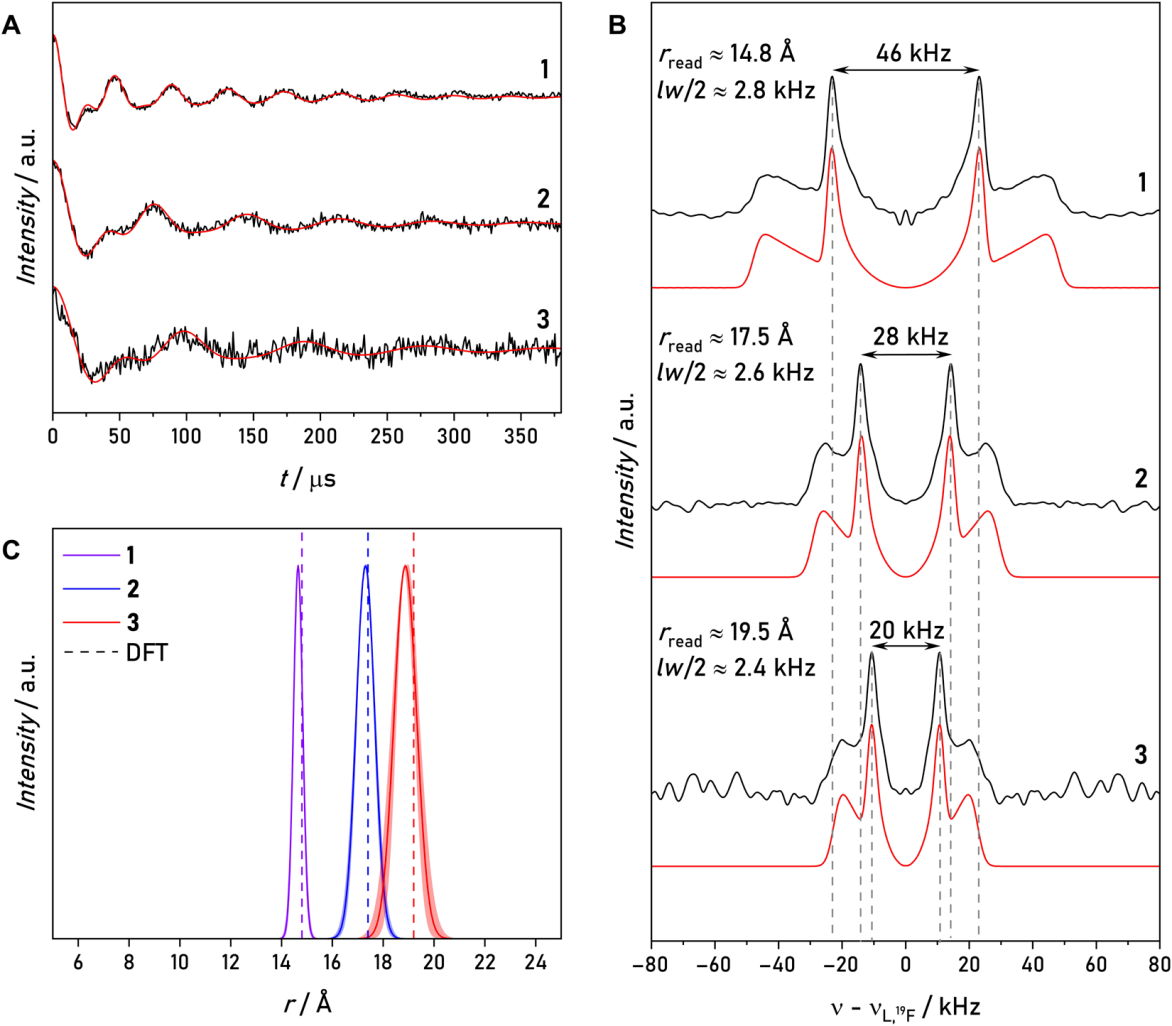
PDHS results for model compounds 1 to 3. (**A**) Sum PDHS time traces of all three model compounds acquired at 50 K. Simulations using a Gaussian distance distribution model are shown as red solid lines. (**B**) ENDOR spectra obtained after Fourier transform of the corresponding time traces. (**C**) Distance distributions obtained from simulations and DFT-predicted distances. Shaded areas indicate the uncertainty of the simulation based on the 95% confidence intervals.

Figure 7 shows the sum PDHS time traces and related Fourier transform spectra of model compounds **1** to **3**. The time traces show a decrease in modulation frequency correlated to the increase in interspin distance. Also, the sensitivity of the experiment reduces for longer interspin distance, although the length of the time trace was kept constant, which is expected due to the 1r6 dependence of the Mims ENDOR intensity (*28*). However, we still observe clear oscillations at interspin distances of ~20 Å. We use also here high sample concentrations (400 μM) to obtain high SNRs for data analysis. The damping of the time traces appears similar, suggesting a similar line broadening mechanism of the dipolar spectra. The splitting in the dipolar Pake pattern can be related to the point-dipole interspin distance. We observe *T*_read_ values of *T*_read,1_ = 46 ± 0.3 kHz, *T*_read,2_ = 28 ± 0.3 kHz, and *T*_read,3_ = 20 ± 0.3 kHz, which result in distances of *r*_1_ = 14.8 ± 0.1 Å, *r*_2_ = 17.5 ± 0.2 Å, and *r*_3_ = 19.5 ± 0.2 Å, in excellent agreement with the DFT-predicted values (Fig. 2 and Table 1).

This study reports a clear hyperfine splitting for a ^19^F ENDOR distance around 2.0 nm. It is important to note that, for model compound **3** with a 19.5-Å distance, FD ENDOR, even after prolonged measurement time, was not able to resolve any splitting at the *g_z_* observer position (fig. S11). In contrast, DTF-TD ENDOR and PDHS resolve a splitting in their respective ENDOR spectra, highlighting their superior sensitivity and resolution. Similar to model compound **1**, neither DTF-TD nor FD ENDOR of compounds **2** and **3** can resolve the principal axis values of the Pake pattern (fig. S12).

**Fig. 8.**
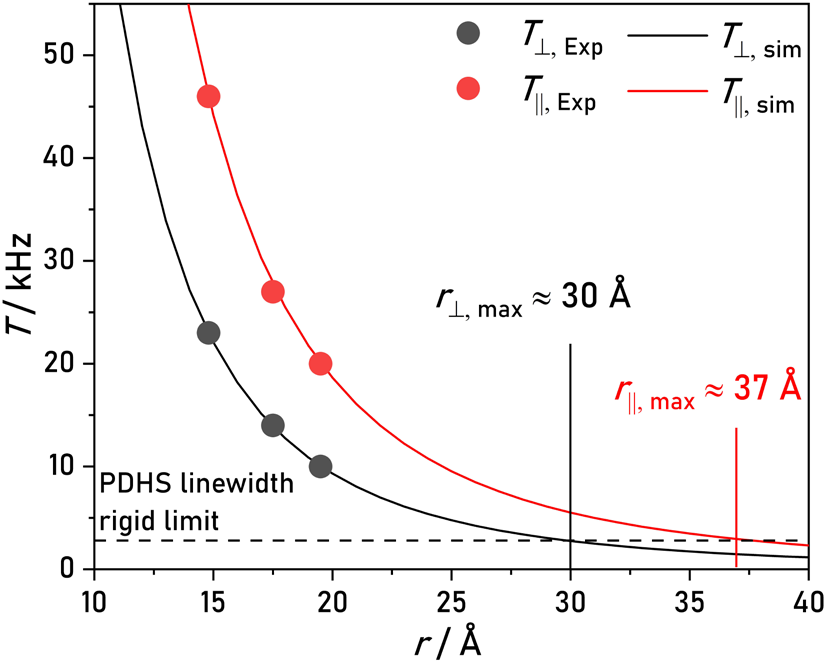
Accessible distance range of PDHS. Simulated dipolar coupling constants $T_{⊥}$ and $T_{∥}$ as a function of a point-dipole interspin distance (Eq. 3; black and red solid lines). Experimental values are shown as red and black dots. Half of the experimental PDHS linewidth of model compound **1** is indicated as horizontal dashed line corresponding to the observed linewidth in FD ENDOR. The expected upper distance limit of PDHS using $T_{⊥}$ and $T_{∥}$ is indicated. Errors in distances and *T* are considered within the size of the used symbols.

Looking at the PDHS time traces and corresponding ENDOR spectra for compounds **1** to **3**, we expect that even much smaller HFCs (i.e., longer interspin distances) could be resolved using PDHS. Figure 8 shows computed HFCs based on the point-dipole model in Eq. 3 as a function of interspin distance. Considering that the observed linewidth of compounds **1** to **3** represents a rigid limit for narrow distance distributions, we can estimate the accessible distance range. We take also into account the observation of the double frequency in PDHS, thus half of the experimental linewidth is marked in the plot. According to this, the resolution limit of PDHS should extend to around 30 Å, effectively doubling the current distance range with FD ^19^F ENDOR (*18*).

Assuming that the linewidth in PDHS is mainly dictated by the distance distribution in the specific molecule, the dipolar signal obtained in PDHS can then be described similarly to other dipolar EPR techniques like DEER (*35*). Neglecting intermolecular interactions, the PDHS time trace can be described by Eq. 5

$$V\left(t,\tau \right)=\int_{0}^{\infty }K\left(r,t,\tau \right)P\left(r\right)\mathrm{dr}$$ (5)

Here, P(r)  is the distribution of interspin distances and K(r,t,τ) the dipolar kernel that describes the connection between the TD signal and the distance distribution. The task is to find the distance distribution P(r) that produces the measured data V(t,τ) . This ill-posed problem can be solved by using a Gaussian distribution model and a least-square fitting algorithm (for details, see the Supplementary Materials)

$$P\left(r\right)=\text{exp}\left[-\frac{{\left(r-\langle r\rangle \right)}^{2}}{2\sigma^{2}}\right]$$ (6)

Here, 〈r〉 is the mean distance and σ is the SD. The results from the simulations are compared with distances obtained from DFT calculations in Fig. 7 and Table 1. From the fitting algorithm, we extracted the 95% confidence intervals.

**Fig. 9.**
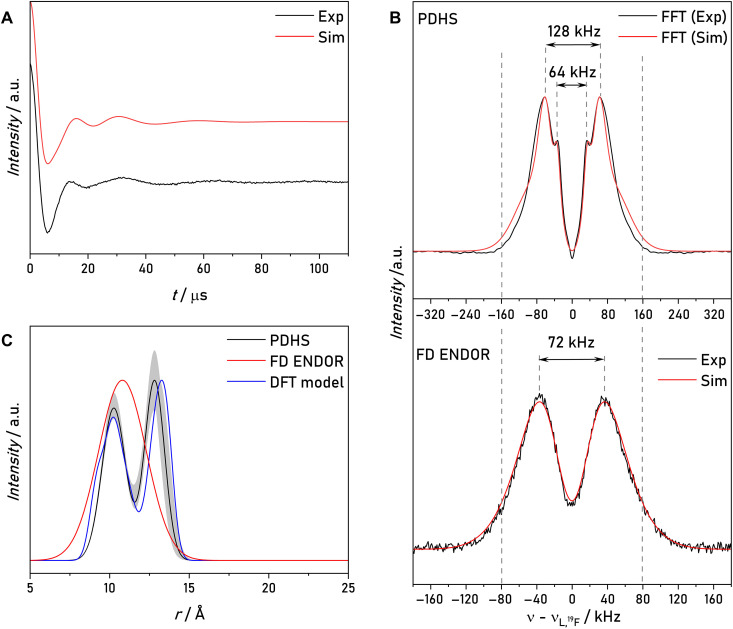
PDHS and FD ENDOR experiments of model compound 4. Simulations are shown as red solid lines. (**A**) Sum PDHS time trace of the model compound **4** and simulation. (**B**) PDHS ENDOR spectrum and simulation using a bimodal Gaussian model. FD ENDOR spectrum and simulation corresponding to a mono-Gaussian distance distribution. (**C**) Distance distributions obtained from simulations and DFT-predicted distance distribution. The shaded area indicates the uncertainty of the simulation based on the 95% confidence intervals.

To examine the case of a flexible spin label with a more complex distance distribution, we conducted FD ENDOR and PDHS experiments on model compound **4**. The FD ENDOR spectrum (Fig. 9) shows a single splitting with a coupling constant of ~72 kHz. To simulate and fit the FD ENDOR spectrum, one might assume a mono-Gaussian distance distribution and an additional linewidth parameter to account for line broadening effects. The procedure leads to a good, misleading agreement between the simulated and experimental FD ENDOR spectrum using a Gaussian distribution ( 〈r〉 = 10.8 Å, σ = 1.5 Å) and a linewidth parameter *lw* = 31 kHz. However, the enhanced resolution of PDHS revealed an additional splitting in the spectrum, thereby excluding the possibility of a mono-Gaussian distance distribution. Using the PDHS traces, we fitted a distance distribution using a bimodal Gaussian. The result is supported by the distance distribution obtained from a conformational analysis of model compound **4** (fig. S13), which revealed 21 relevant conformers. To extract a distance distribution from the ensemble, 21 Gaussian distributions centered at the individual interspin distances of the conformers were summed up to yield the final distance distribution shown in Fig. 9. Additional details are provided in the Supplementary Materials. We could simulate the FD ENDOR spectrum using the same bimodal Gaussian distribution (fig. S14).

## DISCUSSION

In this study, we presented the development of ENDOR nuclear sublevel coherence spectroscopy that increased the current limit of ENDOR sensitivity and resolution by one order of magnitude. DTF-TD ENDOR enabled ENDOR measurements at sample concentrations as low as 2 μM, thus far unattainable with classical FD ENDOR. This enhanced sensitivity paves the way for investigating larger biomolecules under more physiological conditions and reaches the regime of other biophysical methods such as cryo–electron microscopy. Although we demonstrated the application of the method for ^19^F, the higher sensitivity could be advantageous for the detection of many other target nuclei (such as ^13^C and ^31^P). We presented the previously unidentified PDHS pulse sequence that directly detects nuclear echo hyperfine modulations. Similar to pulsed dipolar spectroscopy (PDS), PDHS uses a nuclear spin echo to refocus line broadening interactions such as NDCs, which limit the resolution of FD and DTF-TD ENDOR. An exception might be homonuclear dipolar coupling, which should not be refocused at the time point of the nuclear spin echo. Investigations in this direction are ongoing. The increased resolution of PDHS extends the theoretical distance limit of ^19^F ENDOR distance measurement up to about 3 nm, almost doubling the current reported distance limit. PDHS also offers the opportunity of extracting distance distributions from the acquired time traces. We note that, although demonstrated here for ^19^F ENDOR, PDHS is not limited to ^19^F but should be applicable to other magnetic nucleus of interest. Moreover, we do not see restrictions to particular EPR frequencies, as long as hardware to produce short RF pulses is available. This would offer more opportunities in the choice of samples as the overlap of ^1^H and ^19^F resonances would be circumvented and therefore deuteration of the nitroxide would not be required. In this context, also the effect of CSA in PDHS sequences will be investigated. We foresee that all experience from electron-electron PDS in spin labeling and data analysis achieved in the past two decades, combined with progress in hardware and shape pulse sequences, might be applied in future to the PDHS, rendering this a powerful tool in many areas of magnetic resonance spectroscopy and structural biology. Also, the accessibility of long hyperfine distances could offer opportunities to study polarization transfer mechanisms in DNP, by detecting HFC along pathways of nuclei involved in polarization transfer. A more systematic investigation of DTF-TD ENDOR and PDHS in biomolecules, using different target nuclei, is in progress. The enhanced sensitivity and resolution of the presented methods will benefit a wide range of structural investigations.

## MATERIALS AND METHODS

### Experimental details

EPR and ENDOR measurements were performed at 50 K using a Bruker E580 X/Q-band spectrometer equipped with a Bruker EN 5107D2 pulse EPR/ENDOR resonator in a CF935 helium gas flow cryostat (Oxford Instruments, Abingdon, UK). A 170-W TWT amplifier (model 187Ka, Applied Systems Engineering, USA) and a 600-W RF amplifier (600A225A Amplifier Research) were used to amplify MW and RF pulses, respectively. For FD ENDOR measurements, MW pulse lengths of 12 ns (π/2) and RF pulse lengths (π) of 200 μs were used. A delay time τ of 3 to 8 μs was used, based on the electronic relaxation times (fig. S1). Shot repetition times (SRTs) of roughly five times *T*_1_ were used, corresponding to about 8 ms for all samples. The RF sweep was performed with stochastic acquisition mode with 1 to 10 shots per point (SPP). The echo integration was performed in a 48- to 100-ns window placed symmetrically around the maximum echo intensity.

For PDHS and DTF-TD ENDOR measurements, MW pulse lengths of 12 ns (π/2) and RF pulse lengths (π/2) of 4 to 6 μs were used based on RF nutation experiments (fig. S2). The frequency of the RF channel was set to the Larmor frequency of the ^19^F nucleus. A delay time $\tau_{\mathrm{RF}}$ of 8 μs was used for all DTF-TD ENDOR experiments, ensuring a short time interval of *T*. In PDHS, a $\tau_{\mathrm{RF}}$ of 426 μs (model compounds **1** to **3**) and 144 μs (model compound **4**) was used. A rather large window was chosen to detect the full decay in the PDHS time trace. A delay of 50 to 200 μs between the last RF pulse and the last MW pulse is needed to avoid spectral artifacts. A delay time τ of 3 μs was used for all measurements. The RF/MW pulse was moved in 240- to 960-ns steps. These values were chosen based on the length of the acquired time trace. For both experiments, an eight-step RF phase cycle [0,0,0]-[0,0,π]-[π,0,0]+[π,0,π]+[0,π,0]-[0,π,π]-[π,π,0]+[π,π,π] was used, based on the phase cycle proposed for TD ENDOR (*28*). The phase cycle removes all modulations or background signals that arise from other sources than the RF channel. All PDHS and DTF-TD ENDOR experiments were recorded with 10 SPP and an SRT of 6 to 8 ms. The echo integration was performed in 12- to 100-ns window placed symmetrically around the maximum echo intensity.

### Sample preparation

Synthesis of the deuterated nitroxide model systems was based on established procedures (*6*, *36*–*39*). Some additional information on the synthesis steps of model compounds **2** and **3** along with analytics is presented in the Supplementary Materials. The samples were prepared in solutions of deuterated DMSO-*d*_6_/glycerol-*d*_8_ (2:3). DMSO (Eurisotop) and glycerol (Sigma-Aldrich) both had a deuteration degree of 99.8% according to the manufacturers. A volume of about 12 μl was filled into quartz capillaries (1.6-mm outer diameter, Wilmad 222 T-RB) and frozen in liquid nitrogen.

### Spin dynamics simulations

Spin dynamics simulation allow for an evaluation of the effect of pulse sequences by numerically calculating the evolution of the spin density matrix during the ENDOR pulse sequence (*24*, *33*, *40*). The simulations have been demonstrated for TD-ENDOR (*24*). Here, the simulation script was extended to DTF-TD ENDOR and PDHS by implementation of the adapted pulse sequences. Treatment of the overall simulation procedure remains as described before. Two spin Hamiltonians are introduced to separate the calculation of EPR resonances from the calculation of ENDOR signals (*24*)

H^S,EPR=H^EZ+H^NZ+H^HF+H^NQ=μBgeBS^−μNgnBI^(N14)+hS^A(N14)I^(N14)+hI^(N14)P(N14)I^(N14) (7)

H^S,ENDOR=H^EZ+H^NZ+H^HF+H^NDC=μBgeB0S^z−μNgnB0(1−σ^zz)I^z(F19)+hS^zAzz(F19)I^z(F19)+∑i=12hDzz,i(Hi1)I^z(F19)I^z,i(Hi1) (8)

Then, the EPR Hamiltonian is diagonalized to calculate the EPR resonances for a set grid of orientations with respect to the magnetic field. For each excited orientation, a weighing factor depending on the MW resonance and excitation profile of the MW pulse is determined (*24*, *41*, *42*) before the actual spin dynamics simulation is started. To include transversal relaxation effects, the simulation is performed in Liouville space (*24*). Starting from an initial spin density matrix $\left({\hat{\rho }}_{0}\approx 1-\frac{B_{0}\mu_{B}g_{e}}{k_{B}T}{\hat{S}}_{z}\right)$ using the Liouville–von Neumann equation for time independent Hamiltonians (*43*)

$$\vec{\rho }\left(t\right)=\text{exp}\left[\left(-i{\hat{\hat{L}}}_{S}+\hat{\hat{R}}\right)t\right]\vec{\rho }\left(0\right)$$ (9)

using the external magnetic field $B_{0}$ , the bohr magneton $\mu_{B}$ , the g factor ( g ), the Boltzmann constant $k_{B}$ , the spin Hamiltonian in Liouville space ${\hat{\hat{L}}}_{S}$ , and the relaxation superoperator $\hat{\hat{R}}$ . Following the stepwise calculation of the evolution of the spin density matrix, the signal intensity is calculated as the expectation value of the ${\hat{S}}_{y}$ operator. The spin Hamiltonian includes additional terms for the MW and RF pulse, when the pulses are applied (*24*, *33*). The relaxation superoperator is dependent on the considered relaxation rates and induces effects on the spin density matrix dependent on the relaxation rates (*24*, *43*). Here, longitudinal relaxation is neglected. Considering a spin system of two spins 1/2, one nucleus and one electron coupled to one another, the relaxation rates of electron ( $R_{2e}$ ), for the nuclear ( $R_{2n}$ ) zero-quantum ( $R_{2\text{zq}}$ ) and double-quantum ( $R_{2\text{dq}}$ ) coherences, can be considered. Here, $R_{2e}$ and $R_{2n}$ are considered for a four-spin system (electron spin, fluorine, and two protons). The relaxation rates introduce a decay in the density matrix elements corresponding to the respective coherences. $R_{2\text{zq}}$ and $R_{2\text{dq}}$ were set to zero. Additional information on spin dynamics simulations is provided in (*40*).

## Supplementary Materials

### This PDF file includes:

Supplementary Text Tables S1 to S6 Figs. S1 to S14 References

Correction (25 August 2025):
Due to a production error, the incorrect Supplementary Materials file was inadvertently used for publication. The updated file includes minor updates to figure captions and two corrected figure references. The Supplementary Materials PDF has been updated. The original version is available here:
