# Supplementary material for: Pulsed dipolar hyperfine spectroscopy for molecular distance measurements in the angstrom to nanometer scale
Source: Sci Adv. 2025 Jul 25;11(30):eady5665. doi: 10.1126/sciadv.ady5665 (PMC12292905; doi:10.1126/sciadv.ady5665)
Supplement: Supplementary file 1 — Supplementary Text Tables S1 to S6 Figs. S1 to S14 References [file sciadv.ady5665_sm.v2.pdf]

CORRECTED 25 AUGUST 2025; SEE BELOW

Science Advances
AAAS

Supplementary Materials for

Pulsed dipolar hyperfine spectroscopy for molecular distance measurements in the angstrom to nanometer scale

Lucca Sielaff *et al.*

Corresponding author: Lucca Sielaff, lucca.sielaff@mpinat.mpg.de;
Marina Bennati, marina.bennati@mpinat.mpg.de

Sci. Adv. **11**, eady5665 (2025)
DOI: 10.1126/sciadv.ady5665

This PDF file includes:

Supplementary Text
Tables S1 to S6
Figs. S1 to S14
References

Correction (25 August 2025): Due to a production error, the incorrect Supplementary Materials file was inadvertently used for publication. The updated file includes minor updates to figure captions and two corrected figure references. The Supplementary Materials PDF has been updated.

Supplementary Text

1. Synthesis of the model compounds

The synthesis of the deuterated nitroxide was performed following previously reported procedures (*24*, *36–39*). Dicyclohexylcarbodiimid (DCC), 4-[(4-Flourophenyl)ethynyl]phenol, and 4-Flourophenylboronic acid were purchased from Sigma-Aldrich. Dimethylaminopyridine (DMAP) was purchased from Acros organics and 4-Hydroxy-4'-iodobiphenyl from fisher scientific. 4"-fluoro-[1,1':4',1"-terphenyl]-4-ol was synthesized as discussed below. Solvents and all remaining chemicals were purchased from either Sigma-Aldrich, Acros organics or TCI chemicals. All chemicals were used as received without further purification. All three model compounds were obtained by Steglich esterifications as reported previously (*6*). The purity of the compounds was assessed via LCMS.

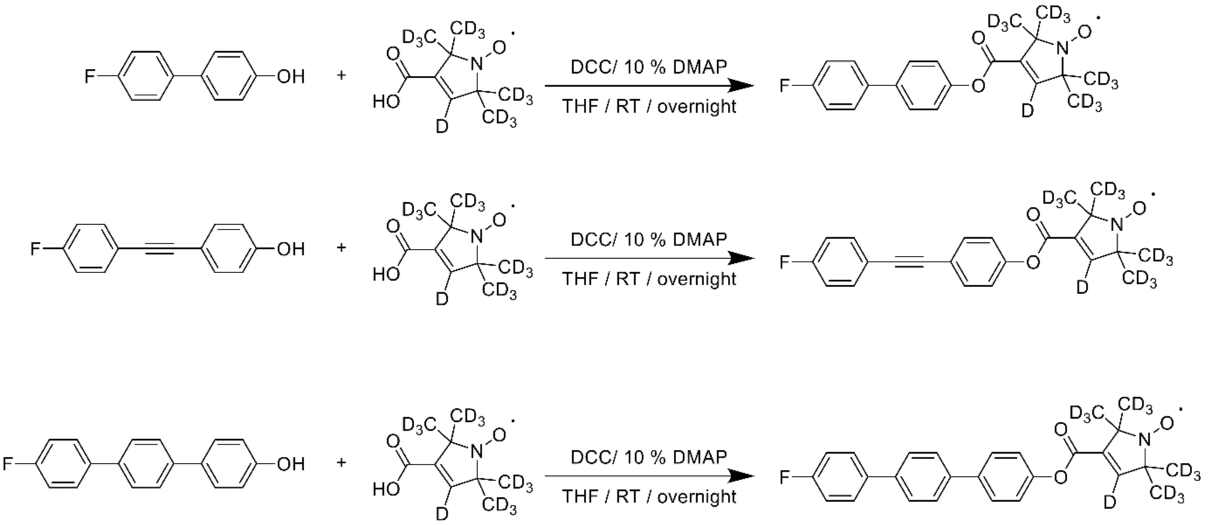

ESI-MS for 1 : *m/z* 356.3 (100 %, $[C_{21}H_8D_{13}FNO_3]^+$, $[M]^+$).
ESI-MS for 2: *m/z* 393.3 (100 %, $[C_{23}H_8D_{13}FNO_3]^+$, $[M]^+$).
ESI-MS for 3: *m/z* 445.3 (100 %, $[C_{27}H_{12}D_{13}FNO_3{}^+$, $[M]^+$).

Synthesis of 4''-fluoro-[1,1':4',1''-terphenyl]-4-ol

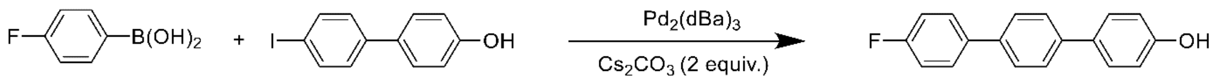

All works were performed in dried Schlenk flasks under nitrogen atmosphere with degassed solutions. Pd_2dBa_3 (27 mg, 0.03 mmol, 0.015 equiv.), 1,3-Dimesitylimidazolium-dichloride (20 mg, 0.06 mmol, 0.06 equiv.) and $CsCO_3$ (1.3 g, 4 mmol, 2 equiv.) were added to a schlenk flask. A solution of 4-Flourophenyl boronic acid (420 mg, 3 mmol, 1.5 equiv.) and 4'-iodo-[1,1'-biphenyl]-4-ol (592 mg, 2 mmol, 1 equiv.) in 18 ml Dioxan was added. The mixture was stirred at 80°C for 2 h and filtered over Celite. The product was purified by column chromatography (1:3:10 / THF,DCM,Hexane). A mixture of product and 4'-iodo-[1,1'-biphenyl]-4-ol was obtained. Therefore, the product was further purified via recrystallisation yielding the product (280 mg, 1.06 mmol, 53 % yield) as a white participate. The purity of the compound was assessed via LCMS.

ESI-MS: *m/z* 264.1 (100 %, $[C_{21}H_8D_{13}FNO_3]^+$, $[M]^+$).

2. DFT Calculations

The starting structure for the DFT optimization at the SVP level was obtained using the chemical graphics program Avogadro (*44*). After initial optimization, the structure was re-optimized two times, first using BP86 functional with the def2-tzvp basis set (*6*) and a second time using wB97X functional with the D4 dispersion correction (*45*). The hyperfine coupling tensor was calculated for the final geometry optimization and then used to calculate the inter-spin distance using equation S1.

$$r = \sqrt[3]{C / T} \tag{S1}$$

where T is the principle axis value of the dipolar part of the hyperfine coupling tensor. For model compounds **1** and **2** small isotropic coupling constants were predicted by ORCA.

$$T_{\text{orca}} = \begin{pmatrix} 2T & & \\ & -T & \\ & & -T \end{pmatrix} + a_{\text{iso}} = \begin{pmatrix} T_1 & & \\ & -T_2 & \\ & & -T_3 \end{pmatrix} \tag{S2}$$

We used the approximation $T = 0.5 \cdot T_1$. The results obtained from the DFT calculations are summarized in Table S1. The corresponding xyz-coordinates of the optimized structures of model compound **1-3** are shown in Table S2-S4.

Table S1. Hyperfine parameters of model compounds **1-3** obtained from DFT calculations.

Sample	T_1 / kHz	T_2 / kHz	T_3 / kHz	a_{iso} / kHz
**1**	45.6	22.8	22.3	0.2
**2**	28.2	14.1	12.7	0.4
**3**	21.2	10.6	10.6	0

Table S2. xyz coordinates of model compound **1**

Atom	x / Å	y / Å	z / Å
F	-5.616914	1.373226	-0.031165
C	-4.256113	1.25614	-0.028728
C	-3.491116	2.307325	-0.523029
C	-3.683641	0.089825	0.467881
C	-2.29185	-0.020034	0.465871
C	-2.100953	2.177913	-0.516828
C	-1.473921	1.016651	-0.02429
C	0.004137	0.889483	-0.022363
C	0.780871	1.450049	-1.055548
C	0.673394	0.202066	1.008255
C	2.064128	0.080151	1.014015
C	2.170833	1.33318	-1.06333
C	2.800023	0.649571	-0.023123
O	4.190399	0.442387	-0.051677
H	0.292068	1.965436	-1.8837
H	2.762059	1.755754	-1.87654
H	0.104216	-0.228723	1.833501
H	2.577488	-0.44734	1.818848
H	-4.31619	-0.717378	0.838408
H	-1.838187	-0.941289	0.834248
H	-1.496053	3.008583	-0.883493
H	-3.975299	3.210508	-0.895637
C	5.011345	1.508807	0.239901
O	4.589792	2.602626	0.577139
C	6.434484	1.151135	0.098846
C	6.903559	-0.046636	-0.298512
C	7.549044	2.142695	0.392994
C	8.400619	-0.108932	-0.356046
N	8.736676	1.276139	0.093341
C	8.921445	-0.352273	-1.786128
C	8.987133	-1.147239	0.619297
C	7.602122	2.588274	1.864556
C	7.553213	3.360136	-0.548427
O	9.938017	1.698441	0.193488
H	6.288288	-0.905447	-0.563368
H	8.612738	-0.987087	1.639165
H	10.083028	-1.067752	0.623088
H	8.711728	-2.159878	0.294623
H	8.496899	0.375129	-2.490804
H	8.648978	-1.364666	-2.114135
H	10.01673	-0.26433	-1.796871
H	7.494041	3.046606	-1.599544
H	8.481182	3.928954	-0.397797
H	6.699795	4.010703	-0.321227
H	7.582945	1.722095	2.539957
H	6.74636	3.236412	2.090987
H	8.527907	3.154333	2.037502

Table S3. xyz coordinates of model compound **2**.

Atom	x / Å	y / Å	z / Å
F	-5.616914	1.373226	-0.031165
C	-4.256113	1.25614	-0.028728
C	-3.491116	2.307325	-0.523029
C	-3.683641	0.089825	0.467881
C	-2.29185	-0.020034	0.465871
C	-2.100953	2.177913	-0.516828
C	-1.473921	1.016651	-0.02429
C	0.004137	0.889483	-0.022363
C	0.780871	1.450049	-1.055548
C	0.673394	0.202066	1.008255
C	2.064128	0.080151	1.014015
C	2.170833	1.33318	-1.06333
C	2.800023	0.649571	-0.023123
O	4.190399	0.442387	-0.051677
H	0.292068	1.965436	-1.8837
H	2.762059	1.755754	-1.87654
H	0.104216	-0.228723	1.833501
H	2.577488	-0.44734	1.818848
H	-4.31619	-0.717378	0.838408
H	-1.838187	-0.941289	0.834248
H	-1.496053	3.008583	-0.883493
H	-3.975299	3.210508	-0.895637
C	5.011345	1.508807	0.239901
O	4.589792	2.602626	0.577139
C	6.434484	1.151135	0.098846
C	6.903559	-0.046636	-0.298512
C	7.549044	2.142695	0.392994
C	8.400619	-0.108932	-0.356046
N	8.736676	1.276139	0.093341
C	8.921445	-0.352273	-1.786128
C	8.987133	-1.147239	0.619297
C	7.602122	2.588274	1.864556
C	7.553213	3.360136	-0.548427
O	9.938017	1.698441	0.193488
H	6.288288	-0.905447	-0.563368
H	8.612738	-0.987087	1.639165
H	10.083028	-1.067752	0.623088
H	8.711728	-2.159878	0.294623
H	8.496899	0.375129	-2.490804
H	8.648978	-1.364666	-2.114135
H	10.01673	-0.26433	-1.796871
H	7.494041	3.046606	-1.599544
H	8.481182	3.928954	-0.397797
H	6.699795	4.010703	-0.321227
H	7.582945	1.722095	2.539957
H	6.74636	3.236412	2.090987
H	8.527907	3.154333	2.037502
F	-5.616914	1.373226	-0.031165
C	-4.256113	1.25614	-0.028728

Table S4. xyz coordinates of model compound **3**.

Atom	x / Å	y / Å	z / Å
O	-6.7862	0.12316	0.30334
N	-5.53597	0.30866	0.11999
C	-4.98276	1.56442	-0.47284
C	-4.50403	-0.71557	0.49177
C	-3.51821	1.24441	-0.44423
C	-5.33861	2.7767	0.40895
C	-5.51652	1.76001	-1.90483
C	-3.24812	0.02916	0.06835
C	-4.75642	-2.00576	-0.3078
C	-4.57428	-0.98757	2.0046
C	-1.905	-0.5587	0.21971
H	-4.68393	-1.82265	-1.38852
H	-5.76335	-2.38041	-0.07732
H	-4.02282	-2.7708	-0.02537
H	-4.38918	-0.07074	2.58063
H	-3.82696	-1.74092	2.28342
H	-5.57227	-1.3715	2.25769
H	-5.25438	0.90669	-2.54436
H	-5.09125	2.67478	-2.33964
H	-6.61017	1.86263	-1.87535
H	-4.94855	2.65338	1.4281
H	-6.43095	2.88668	0.45478
H	-4.91282	3.69075	-0.02678
O	-1.66918	-1.66968	0.66487
O	-0.92043	0.30711	-0.20056
C	0.4124	-0.1398	-0.14525
C	1.23117	0.32106	0.88388
C	0.91515	-0.95897	-1.15521
C	2.26097	-1.325	-1.12499
C	2.57663	-0.04973	0.90074
C	3.11883	-0.88053	-0.09918
H	0.26222	-1.29443	-1.96193
H	0.81592	0.95848	1.66554
H	3.20843	0.29778	1.71948
H	2.65576	-1.94826	-1.92867
C	4.5478	-1.27383	-0.07611
C	5.53878	-0.40459	0.41979
C	4.96621	-2.53259	-0.54972
C	6.3089	-2.90446	-0.52764
C	7.30035	-2.03498	-0.03267
C	6.88166	-0.77584	0.44024
H	5.26076	0.59208	0.76732
H	7.62189	-0.06061	0.80327
H	4.2257	-3.24719	-0.91351
H	6.58629	-3.9017	-0.87394
C	8.7294	-2.42919	-0.00938
C	9.58971	-1.97787	1.0113
C	9.26905	-3.26578	-1.00652
C	10.93697	-2.34241	1.04288
C	10.61369	-3.64114	-0.99303
C	11.42238	-3.1696	0.03563
F	12.73944	-3.53137	0.05718
H	11.59914	-2.00053	1.83891
H	9.19836	-1.34698	1.81058
H	8.6358	-3.61563	-1.82308
H	11.03228	-4.28016	-1.77117
H	-2.77479	1.95072	-0.81139

3. Relaxation measurements

Relaxation times are important for the sensitivity and optimization of ^{19}F ENDOR measurements. Within a variation of ± 10 %, the electronic relaxation times were identical for all model compounds at sample concentrations of 400 µM. The spin-lattice relaxation time of the electron T_{1e} determines the shot repetition rate and was measured using the inversion recovery experiment. The experimental traces were fitted with stretched exponential decays. From these fits the mean relaxation time is calculated and shown in Figure S1. To optimize the τ value, we measured the phase memory time of the stimulated echo as a function of constant time delays T as described previously (*6*). We fitted the experimental traces using mono exponential fitting functions. The resulting phase memory times are shown as a function of *T* in Figure S1.

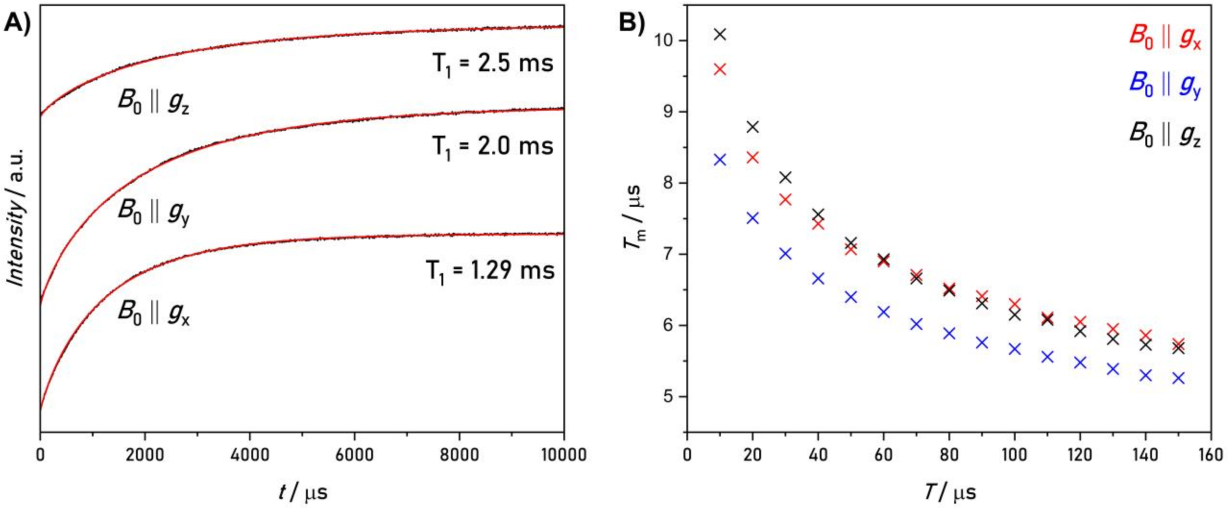

Fig. S1. A) Inversion recovery experiments performed at 50 K for model compound **2** at three different observer positions. **B)** Phase memory times T_M of model compound **2** for different values of *T* at three different observer positions. Values were extracted from the echo decay curve by using mono-exponential fitting functions.

4. RF Nutation

To determine the length of the RF pulses, RF nutation experiments were performed. A representative time trace for model compound **2** is shown in Figure S2. The 600 W RF amplifier was used at 60 % output power and 0 dB attenuation. Subsequently, an RF pulse length of 6 µs for π/2-pulse was used.

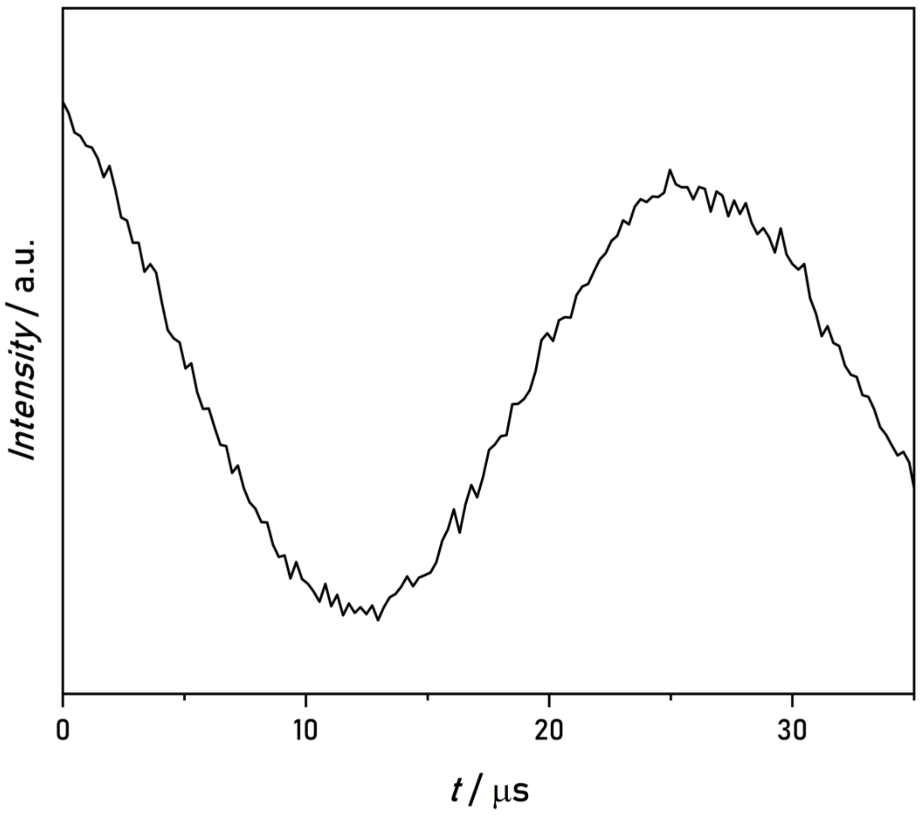

Fig. S2. RF Nutation experiments for model compound **2** performed with a 600 W RF amplifier at 60 % output power and 0 dB attenuation.

5. EPR spectrum and observer positions

A representative echo-detected EPR spectrum of model compound **2** together with an EasySpin (*46*) simulation is shown in Figure S3. The three observer positions for the ENDOR measurements are marked with arrows. The EPR spectrum of **2** is indistinguishable from the EPR spectra of **1, 3** and **4**.

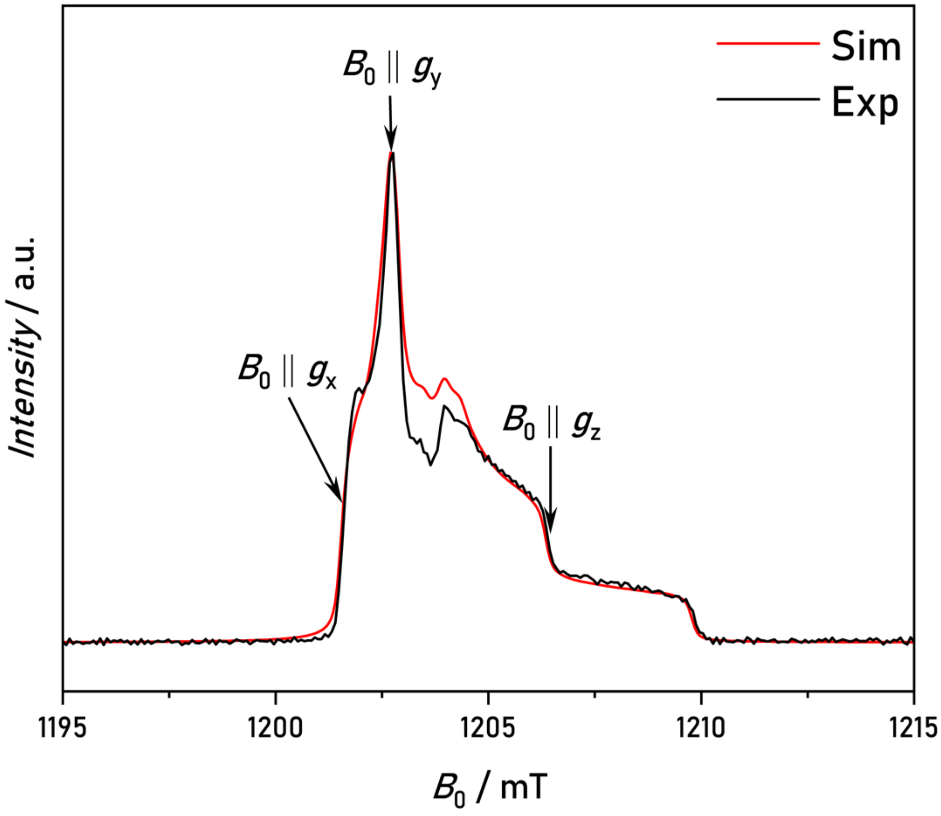

Fig. S3. Echo-detected EPR spectrum of model compound **2**. Simulation parameters: $g_x = 2.00880$, $g_y = 2.00610$, $g_z = 2.00211$, $A_x = 11\ MHz$, $A_y = 11\ MHz$, $A_z = 96\ MHz$ (^{14}N)

6. Total number of scans for all experiments in Figure 3

Table S5. Comparison of total number of scans and acquisition times for all measurements presented in Figure 3. The DTF-TD ENDOR measurement at a sample concentration of 400 μM was recorded with 601 points. All other DTF-TD ENDOR measurements were recorded with 401 points. The FD ENDOR experiments were recorded with 201 points.

Pulse Sequence	Experiment	Shots-per-point	Total number of Scans	Acquisition time
FD ENDOR	50 us RF pulse (400 μM)	1	200	20 min
	200 us RF pulse (400 μM)	1	200	20 min
DTF-TD ENDOR	400 μM	10	16	20 min
	10 μM	10	256	2 h
	2 μM	10	2192	18 h

7. Concentration calibration of model compound **1**

To validate the concentration of our measurement at low sample concentration, we performed echo-detected EPR measurements of 4-Oxo-TEMPO-d_{16} (purchased from Sigma-Aldrich) at different sample concentrations. The corresponding EPR spectra and calibration curve are shown in Figure S4. The spectrum of the sample presented in Figure 3 is shown in blue. Since the experiments were recorded for different acquisition times, we calculated the expected SNR for a measurement time of 64 min using equation S3.

$$SNR_{t_{\mathrm{ref}}} = SNR_{t_{\mathrm{acq}}} \cdot \sqrt{\frac{t_{\mathrm{ref}}}{t_{\mathrm{acq}}}} \qquad \text{(S3)}$$

where t_{ref} is the reference measurement time and t_{acq} is the acquisition time of the experiment. We calculated the concentration of our sample based on the calibration curve, resulting in a concentration of (2.3 ± 0.1) µM.

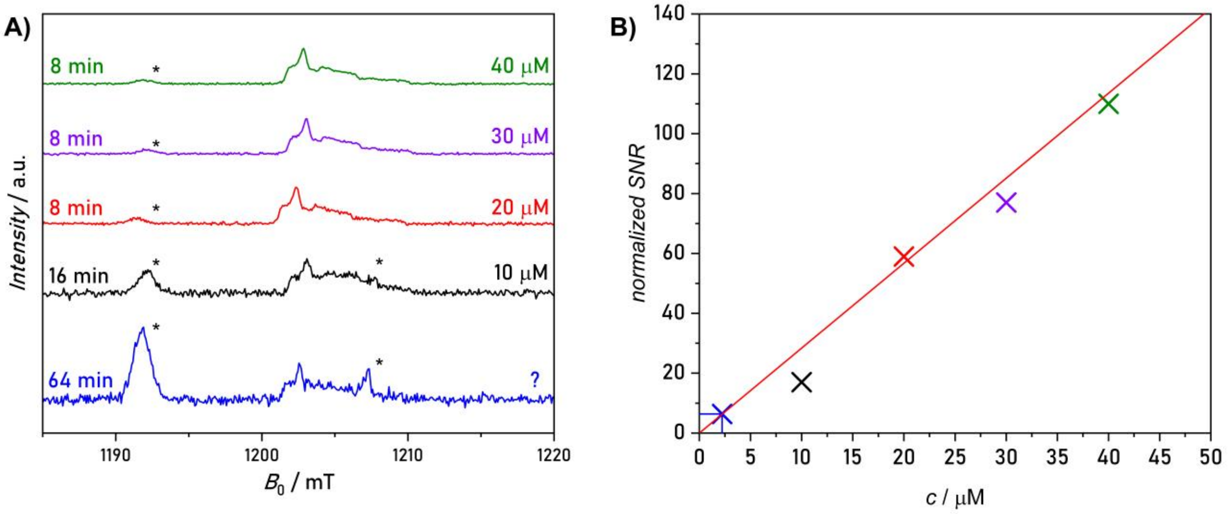

Fig. S4. A) Echo-detected EPR spectra of 4-Oxo-TEMPO-d_{16} with different sample concentrations and sample of model compound **1** with unknown concentration (blue). Asterisks indicate resonator background signal. The relative intensity of the sample and the resonator background signal is visibly changing by lowering the concentration. **B)** normalized SNR as a function of sample concentration. The solid red line indicates the fitted calibration curve. The blue cross marks the observed SNR of model compound **1**.

8. Signal-to-noise ratio calculation for PDHS, DTF-TD ENDOR and FD ENDOR.

We estimated the noise of each experiment by calculating its standard deviation using a filtered version of the experiment (*42*). Since the experiments were recorded for different acquisition times, we calculated the expected SNR for a measurement time of 16 h using equation S3. The resulting SNRs are presented in Table S6.

Table S6. Comparison of normalized SNRs for model compound **1-3** at three observer positions in the EPR line. The SNR was corrected using equation S3 to account for different acquisition times. The asterisk marks a FD ENDOR measurement with a 50 μs RF pulse. All other FD ENDOR measurements were performed with 200 μs RF pulse. The estimated error of the calculated SNRs is ± 5 %.

Model compound	Position	normalized SNR<br>FD ENDOR	 DTF-TD ENDOR	 PDHS
	g_x	15	145	16
**1**	g_y	26 / 72*	161	28
	g_z	15	107	23
	g_x	7	53	10
**2**	g_y	14	46	36
	g_z	6	37	16
	g_x	15	51	12
**3**	g_y	24	70	17
	g_z	5	15	6

9. Product operator formalism of PDHS

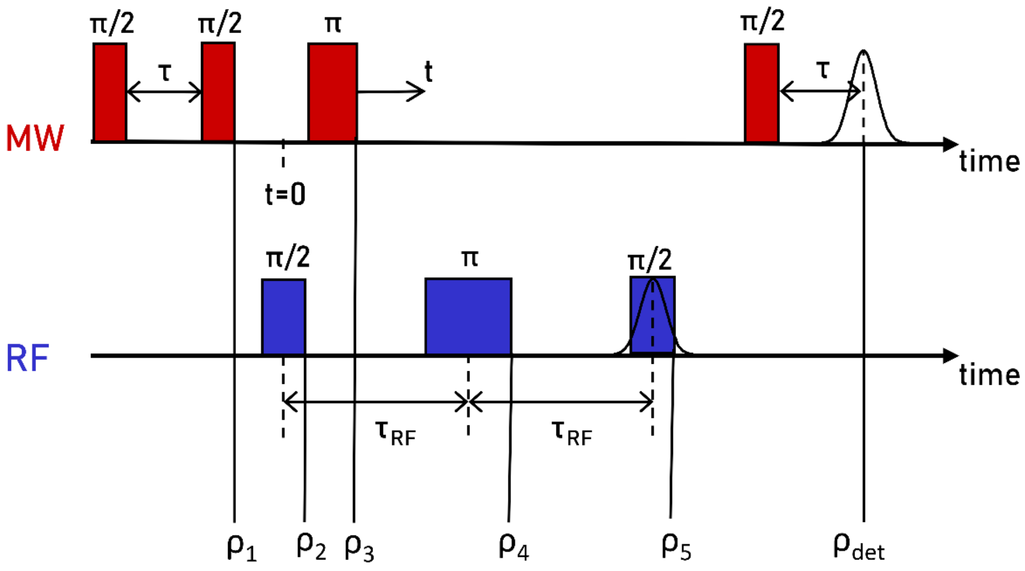

Fig. S5. PDHS sequence and timings used to calculate the time evolution of the density operator ρ.

To predict the effect of the MW pulse on the stimulated echo signal, we employ the product operator formalism for ENDOR according to Gemperle & Schweiger (*27*). Figure S5 shows the PDHS pulse sequence and corresponding time intervals, under which the density matrix evolves under a specific Hamiltonian. For the free evolution steps, we consider the hyperfine Hamiltonian of a two-spin ½ systems, in the high-field approximation and in the doubly rotating given in main text, equation 1. All MW and RF pulses are considered as non-selective. During the preparation step on the MW channel, two π/2 microwave pulses with x-phase are separated by the delay time τ. This part is analogous to the Mims ENDOR sequence and leads to the density operator $\hat{\rho}_1$.

$$\hat{\rho}_{\text{eq}} = -\hat{S}_z \xrightarrow{\frac{\pi}{2}\hat{S}_x} \xrightarrow{\Delta\omega_S\tau\hat{S}_z} \xrightarrow{\tau\frac{A}{2}2\hat{S}_z\hat{I}_z} \xrightarrow{\frac{\pi}{2}\hat{S}_x} \hat{S}_z\cos(\Delta\omega_S\tau)\cos\left(\frac{A}{2}\tau\right) -2\hat{S}_z\hat{I}_z\sin(\Delta\omega_S\tau)\sin\left(\frac{A}{2}\tau\right) = \hat{\rho}_1 \qquad \text{(S4)}$$

where we have omitted electron coherence terms that decay with T_M and do not contribute to the ENDOR signal. After the first RF π/2 pulse we obtain $\hat{\rho}_2$:

$$\hat{\rho}_1 \xrightarrow{\frac{\pi}{2}\hat{I}_x} \hat{S}_z\cos(\Delta\omega_S\tau)\cos\left(\frac{A}{2}\tau\right) + 2\hat{S}_z\hat{I}_y\sin(\Delta\omega_S\tau)\sin\left(\frac{A}{2}\tau\right) = F_1\hat{S}_z + F_2\cdot 2\hat{S}_z\hat{I}_y = \hat{\rho}_2 \qquad \text{(S5)}$$

To better illustrate the subsequent steps, we have collected the pre-factors of the operators in $\hat{\rho}_2$ as F_1 and F_2, which remain constant during the mixing part of the sequence. In the following steps, we will omit the effect of the nuclear offset$\Delta\omega_I$, since we are detecting a nuclear spin echo that refocuses this. After free evolution and the microwave π pulse we obtain $\hat{\rho}_3$ as:

$$\hat{\rho}_2 \xrightarrow{t\frac{A}{2}2\hat{S}_z\hat{I}_z} \xrightarrow{\pi\hat{S}_x} -F_1\hat{S}_z - F_2\left[2\hat{S}_z\hat{I}_y\cos\left(\frac{A}{2}t\right) + \hat{I}_x\sin\left(\frac{A}{2}t\right)\right] = \hat{\rho}_3 \tag{S6}$$

The microwave π pulse causes a change in the sign of the $2\hat{S}_z\hat{I}_y$ operator, which will lead to a modulation of the electron spin echo.

$$\hat{\rho}_3 \xrightarrow{(\tau_{\mathrm{RF}}-t)\frac{A}{2}\hat{S}_z\hat{I}_z} \xrightarrow{\pi\hat{I}_x} -F_1\hat{S}_z - F_2\left[-2\hat{S}_z\hat{I}_y\cos\left(\frac{A}{2}t\right)\cos\left(\frac{A}{2}(\tau_{\mathrm{RF}}-t)\right) - \hat{I}_x\cos\left(\frac{A}{2}t\right)\sin\left(\frac{A}{2}(\tau_{\mathrm{RF}}-t)\right)\right.$$
$$\left. +\hat{I}_x\sin\left(\frac{A}{2}t\right)\cos\left(\frac{A}{2}(\tau_{\mathrm{RF}}-t)\right) - 2\hat{S}_z\hat{I}_y\sin\left(\frac{A}{2}t\right)\sin\left(\frac{A}{2}(\tau_{\mathrm{RF}}-t)\right)\right] = \hat{\rho}_4 \tag{S7}$$

After evolution for a second period τ_{RF}, we can summarize all terms in $\hat{I}_x$ and $2\hat{S}_z\hat{I}_y$. Neglecting non-detectable terms in $\hat{I}_x$ this reduces to:

$$\hat{\rho}_4 \xrightarrow{\tau_{\mathrm{RF}}\frac{A}{2}2\hat{S}_z\hat{I}_z} -F_1\hat{S}_z - F_2\left[-2\hat{S}_z\hat{I}_y\cos\left(2\cdot\frac{A}{2}t\right) + \hat{I}_x\cos\left(2\cdot\frac{A}{2}t\right)\right]$$
$$\xrightarrow{\frac{\pi}{2}\hat{I}_x} -F_1\hat{S}_z + F_2\left[2\hat{S}_z\hat{I}_z\cos(At)\right] = \hat{\rho}_5. \tag{S8}$$

In the last step we re-introduce the factors F_1 and F_2 and neglect all magnetization that is not detectable.

$$\hat{\rho}_5 \xrightarrow{\frac{\pi}{2}\hat{S}_x} \xrightarrow{\tau\hat{S}_z} \xrightarrow{\tau\frac{A}{2}\hat{S}_z\hat{I}_z} \hat{S}_y\left[\cos^2(\Delta\omega_S\tau)\cos^2\left(\frac{A}{2}\tau\right)\right.$$
$$\left. +\sin^2(\Delta\omega_S\tau)\sin^2\left(\frac{A}{2}\tau\right)\cdot\cos(At)\right] = \hat{\rho}_{\mathrm{det}} \tag{S9}$$

We note that, in contrast to the Mims ENDOR treatment (*27*), the sign of the $\hat{S}_y$ operator at detection is here positive, due to the additional MW π pulse. The echo intensity for the average overall resonant offsets $\Delta\omega_S$, neglecting *t*-independent terms, results as:

$$I(t) = \frac{1}{2}\sin^2\left(\frac{A}{2}\tau\right)\cdot\cos(At)\ . \tag{S10}$$

10. Dead-time-free detection in PDHS

Despite the similarity of PDHS and 3-pulse DEER (*47*) a main difference between the two pulse sequences is the possibility to detect PDHS time traces dead-time-free. This is possible because the π/2 RF pulse and the π MW pulse are allowed to overlap. Figure S6 shows two measurements of model compound **1** at g_y position where the time interval t_0 (Fig. S6) was set to 30 µs and 100 µs. The recorded time traces show that a dead-time-free detection of PDHS traces is possible. The zero point of the time traces is chosen by the length of the interval t_0. For the measurements shown in the main text a short t_0 value of 3 µs was used to maximize the signal intensity.

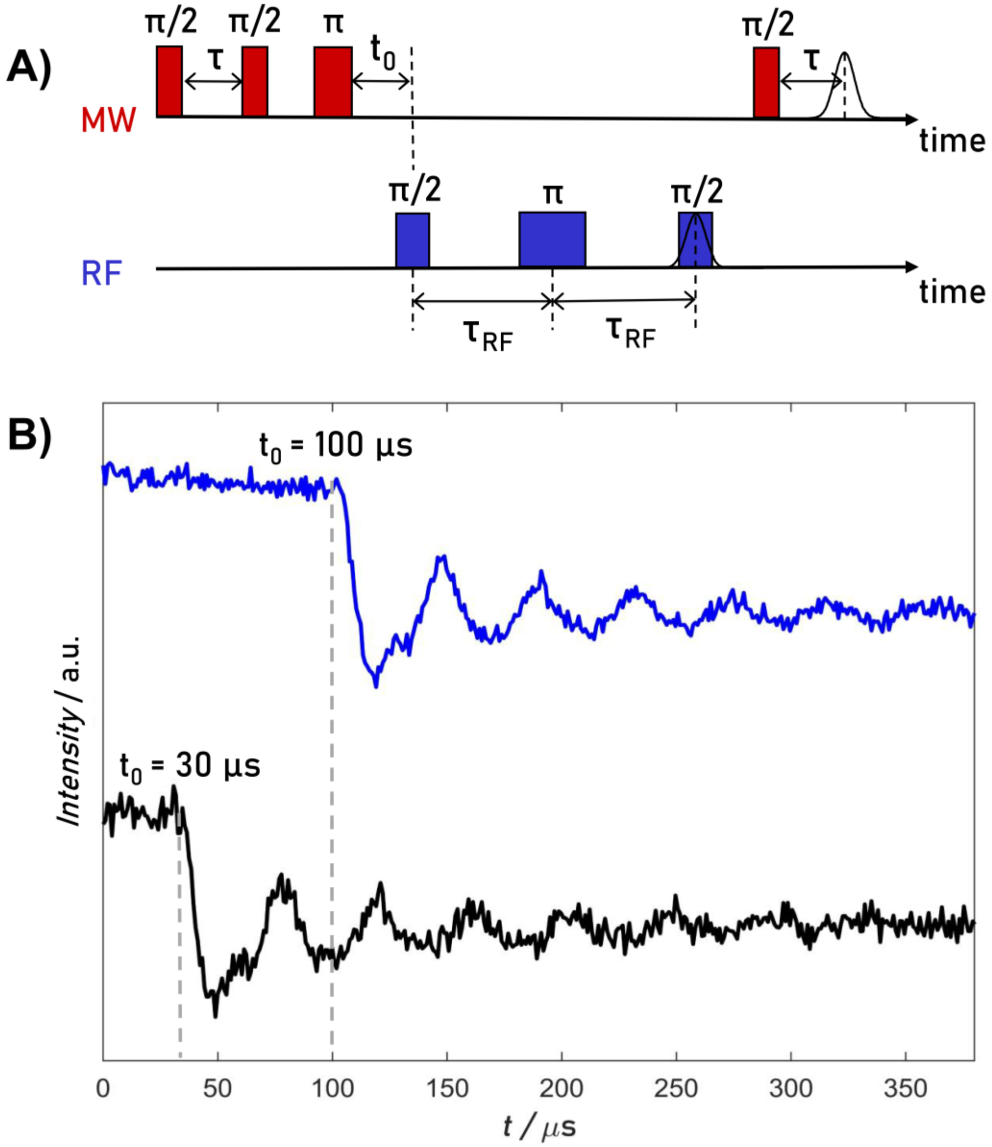

Fig. S6. A) Schematic PDHS pulse sequence including the time interval $\boldsymbol{t_0}$. **B)** PDHS time traces of model compound **1** acquired at g_y position with different values of $\boldsymbol{t_0}$. All other time intervals and pulse lengths were used analogously to the measurements presented in the main text. The blue trace was recorded with twice the number of scans compared to the black trace.

11. Pake pattern and blind spot function

Figure S7 shows a simulated Pake pattern of model compound **1** without distortions caused by the blind spot function (see equation 2). After multiplication with the blind spot function a distorted Pake pattern is observed, which is attenuated towards zero frequencies in the center. Towards the parallel component an increased in intensity is observed.

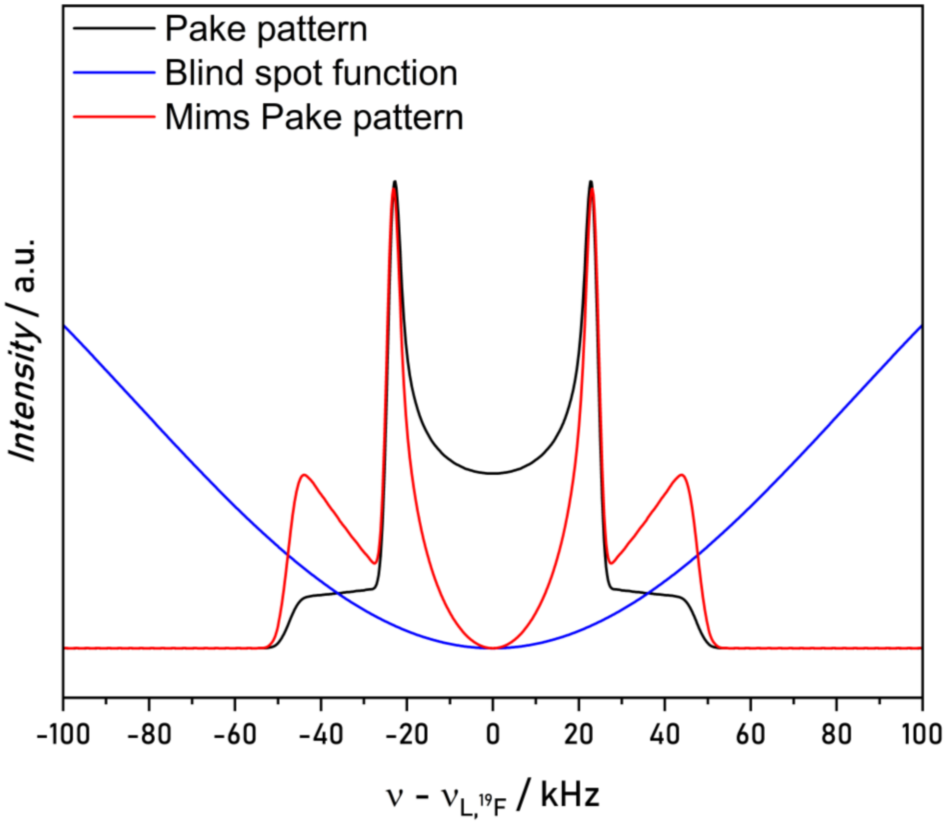

Fig. S7. Simulations of ideal Pake patterns for model compound **1**. The solid black line shows the undistorted Pake pattern. Multiplication with the blind spot function (solid blue line) leads to the observed natural line shape in PDHS (solid red line).

12. PDHS measurements at lower concentration

To show the feasibility of PDHS measurements at lower sample concentrations we performed PDHS experiments of model compound **1** with a sample concentration 100 μM. The resulting time trace and spectrum is compared to the data with sample concentrations of 400 μM in Figure S8.

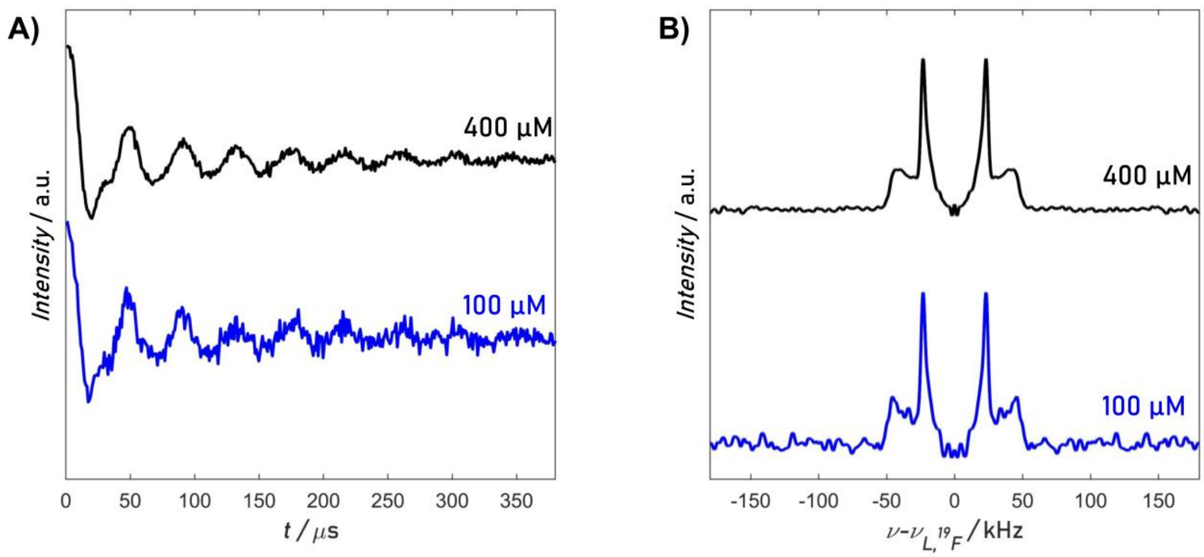

Fig. S8. **A)** Sum PDHS time traces of model compound **1** with sample concentrations of 400 μM and 100 μM. **B)** ENDOR spectra acquired after Fourier transform of the recorded time traces.

13. DFT-optimized structure of model compound **1**

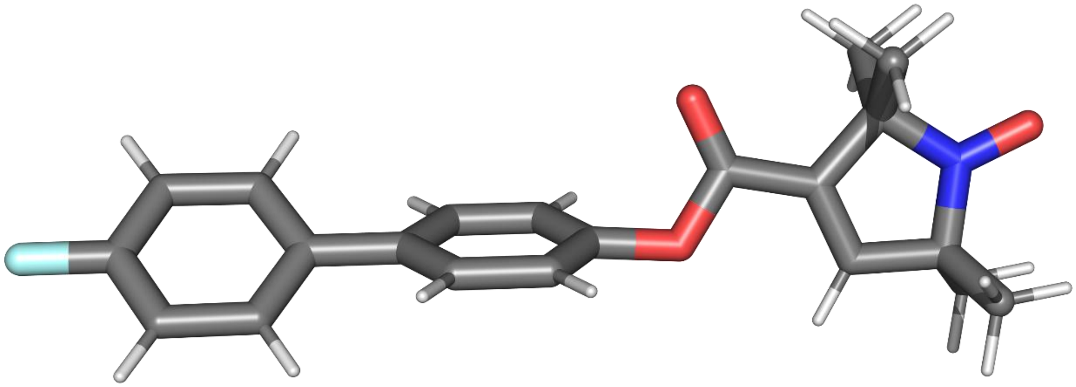

Fig. S9. DFT-optimized structure of model compound **1**.

14. Additional spin dynamics simulations

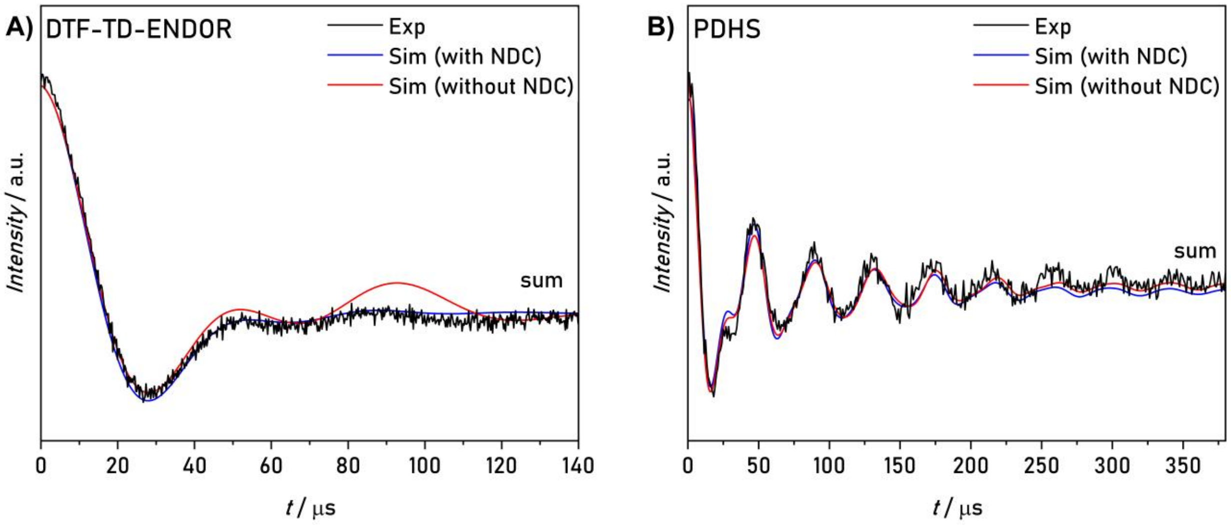

Fig. S10. **A)** Spin dynamics simulation of DTF-TD ENDOR trace of model compound **1**. **B)** Spin dynamics simulation of PDHS trace of model compound **1**. Simulations were performed with and without nuclear dipolar coupling and multiplied with equivalent exponential functions to account for the distance distributions of the sample. All simulation parameters are given in Figure 6 in the main text.

15. Comparison of FD, DTF-TD-ENDOR and PDHS for model compound **2** and **3**

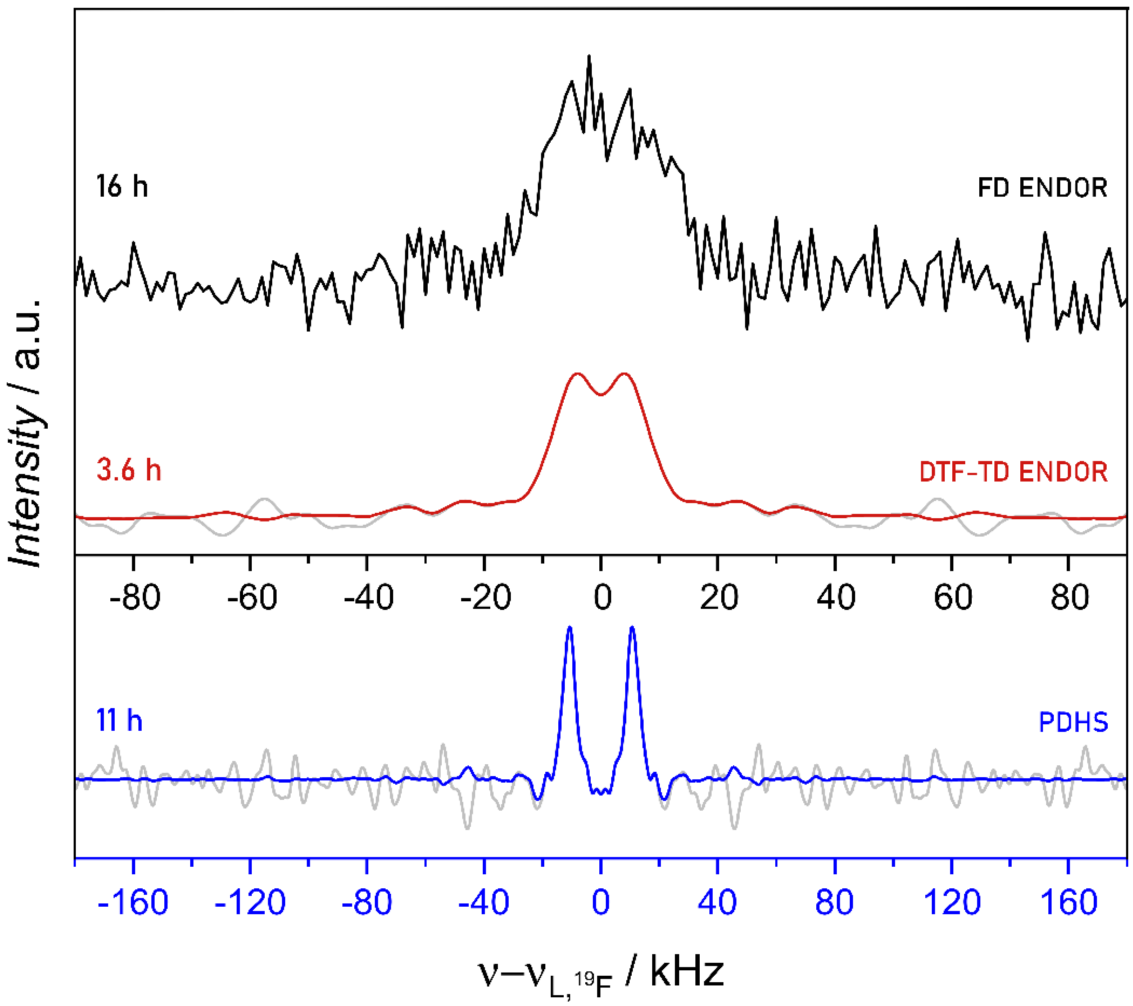

Fig. S11. ENDOR spectra acquired with FD (200 μs RF pulse), DTF-TD ENDOR and PDHS for model compound **3** at g_z position with their respective acquisition times. Grey lines represent the raw data after Fourier transform. Blue and red solid lines show the Fourier transform after smoothing the time trace with a Savitzky-Golay filter. Experimental parameters and Fourier transform procedure are shown in and the experimental details section and Figure 5.

Figure S12 shows the ENDOR spectra acquired with FD, DTF-TD ENDOR and PDHS for model compound **2** and **3**. As discussed in the main text the spectra and time traces of three observer positions were summed up after they had been normalized to their respective EPR intensity.

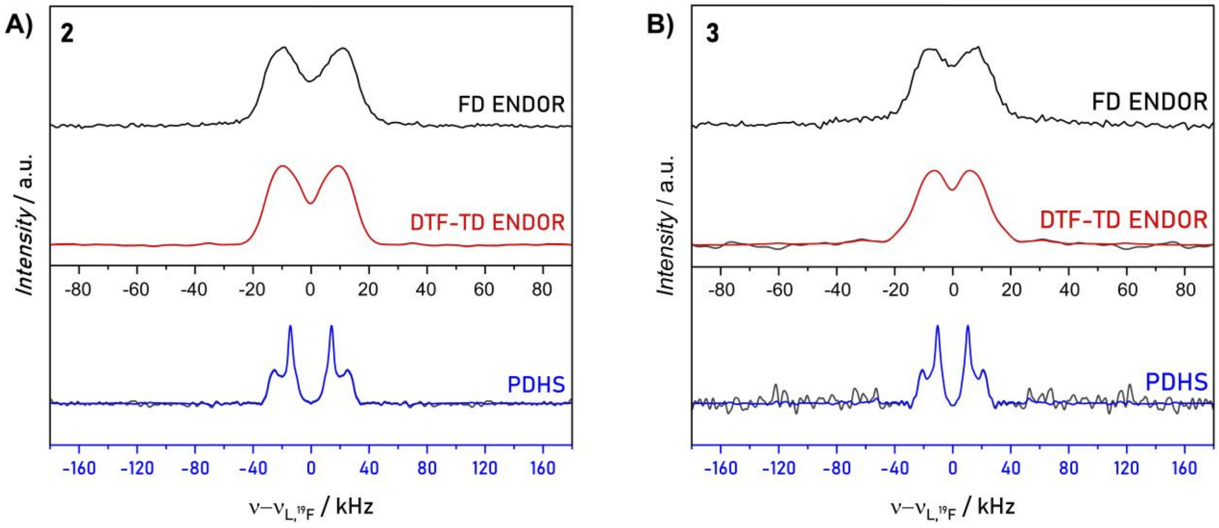

Fig. S12. A) ENDOR spectra acquired with FD (200 µs RF pulse), DTF-TD ENDOR and PDHS for model compounds **2**. **B)** ENDOR spectra acquired with FD (200 µs RF pulse), DTF-TD ENDOR and PDHS for model compounds **3**. Grey lines represent the raw data after Fourier transform. Blue and red solid lines show the Fourier transform after smoothing the time trace with a Savitzky-Golay filter. Experimental parameters and Fourier transform procedure are shown in the experimental details section and Figure 5.

16. Extracting distance distributions from PDHS data

The dipolar signal obtained in PDHS can be described similarly to other dipolar EPR techniques like DEER (*35*). Neglecting inter-molecular interactions, the time trace in PDHS is given as:

$$V(t) = \int_0^{\infty} K(r,t,\tau)P(r)dr \qquad \text{(S11)}$$

Here $P(r)$ is the distribution of inter-spin distances and $K(r,t)$ the dipolar kernel that describes the connection between the time domain signal and the distance distribution. The dipolar kernel function for PDHS under the assumptions of no orientation selection and infinite excitation bandwidth can be formulated in the following way:

$$K(r,t,\tau) = \sum_{\theta=0}^{\pi/2} B(\nu(\theta,r),\tau) \cdot \sin(\theta) \cdot [\cos(2\pi \cdot \nu(\theta,r) \cdot t) + \cos(-2\pi \cdot \nu(\theta,r) \cdot t)] \qquad \text{(S12)}$$

with the blindspot function $B(\nu(\theta,r),\tau)$

$$B(\nu(\theta,r),\tau) = 0.5 \cdot \sin^2\left(2\pi \frac{\nu(\theta,r)}{2}\tau\right) \qquad \text{(S13)}$$

where τ is the separation between the two microwave preparation pulses in the pulse sequence and $\nu(\theta,r)$ the hyperfine coupling frequency given as:

$$\nu(\theta,r) = \frac{\mu_0}{4\pi h}\left(\frac{g_e g_n \mu_B \mu_n}{r^3}\right) \cdot (1 - 3\cos^2(\theta)) = \frac{C}{r^3}(1 - 3\cos^2(\theta)) \qquad \text{(S14)}$$

where *C* is the dipolar constant calculated using μ_0 the vacuum permeability, *h* the Planck constant, the g-factors and Bohr/nuclear magnetons of the radical and nucleus, respectively. The dipolar angle θ describes the orientation of the inter-spin distance vector towards the external magnetic field. The main difference in the equation of the dipolar kernel, compared to other dipolar EPR experiments is the blindspot function $B(\nu(\theta,r),\tau)$ that depends on the experimental parameter τ. To simulate a PDHS time trace a distance vector *r* and a time vector *t* are defined that generate the dipolar kernel $K(r,t,\tau)$. Afterwards equation S15 is solved using a Gaussian model for $P(r)$.

$$G = \mathrm{argmin}_P\{\| \boldsymbol{KP} - \boldsymbol{V}_{\mathrm{Exp}}\|^2\} \qquad \text{(S15)}$$

G is a vector containing all model parameters ($\langle r \rangle, \sigma$) and $\boldsymbol{V}_{\mathrm{Exp}}$ the experimental data. FD ENDOR spectra are simulated in close analogy. However, the dipolar Kernel is extended by a line-broadening Gauss function to account for additional line-broadening interactions.

17. Conformational analysis of model compound **4**

Model compound **4** consists of four rotatable bonds (dihedral angles $\phi_1 - \phi_4$) that influence the ^{19}F-electron distance (see Figure 2). Based on the rotational symmetries around the dihedral angles $\phi_1 - \phi_4$ we expect 3 x 3 x 2 x 2 = 36 non-equivalent local energy minima. This hypothesis was confirmed using constrained surface scans in which the respective dihedrals were incremented in 10° steps using the ORCA software at the BP86/def-tzvp level with dispersion correction and resolution of identity and chain-of-spheres approximation (*48–50*).The found 36 geometries could be categorized into three according to their energies, which were either within 1 kJ/mol to the lowest energy conformer, about 2.5 – 3 kJ/mol above the lowest energy, or about 7 – 8 kJ/mol above the lowest energy. These 12 high energy conformers (7 – 8 kJ/mol) were discarded, as it was expected that they would not be populated to a notable degree under EPR/ENDOR conditions (i.e. frozen solution). For the remaining 24 conformers, another unrestrained optimization at the same level of theory was performed. In three cases belonging to the 3 kJ/mol category, the optimization resulted in a conformational change yielding a redundant conformer at lower energy, meaning that 21 non-redundant conformers were obtained as final result. Based on the calculated energies, 15 conformers were excluded from the original ensemble, because they are thermally inaccessible at temperatures of 200 K. The resulting 21 possible conformations of the model compound are shown in Figure S13. To extract a distance distribution from the ensemble 21 Gaussian distributions centered at the individual inter-spin distances of the conformers were summed up to yield the final distance distribution shown in Figure 9. Each Gaussian distribution had a variance of σ = 0.4.

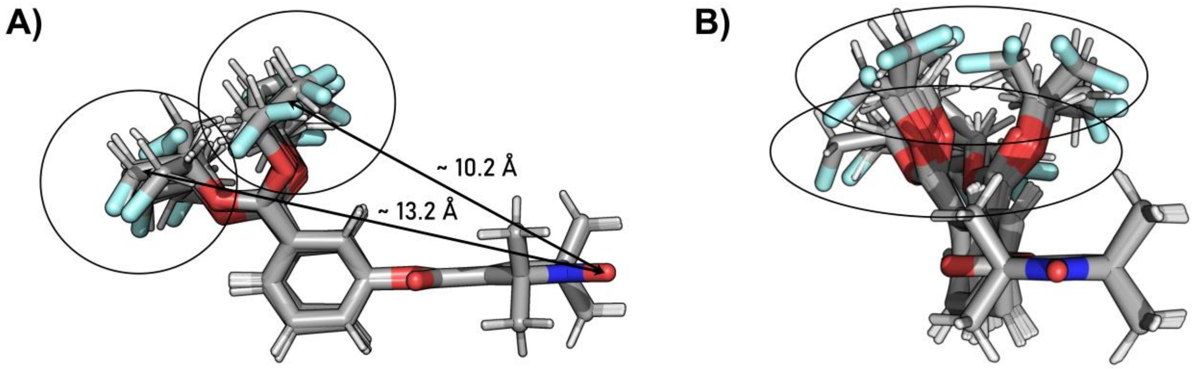

Fig. S13. Ensemble of 21 relevant conformers of model compound **4. A)** Side view of the conformational ensemble and average inter-spin distance of the marked fluorine nuclei. **B)** Front view of the ensemble.

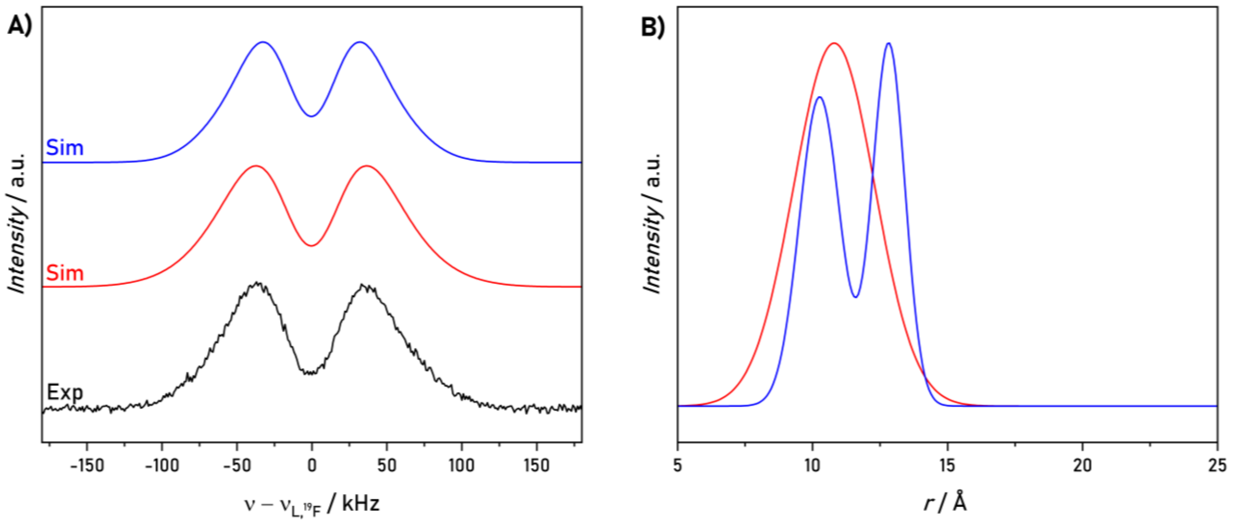

Fig. S14. A) FD ENDOR spectrum and simulations of model compound **4**. The experimental spectrum is shown in black. Simulated FD ENDOR spectra using a bimodal or unimodal Gaussian distribution are shown in blue and red, respectively. **B)** Distance distributions used for the simulation.

REFERENCES AND NOTES

1. D. Goldfarb, S. Stoll, Eds., *EPR Spectroscopy: Fundamentals and Methods*, eMagRes Books (Wiley, 2018).

2. V. K. Michaelis, R. G. Griffin, B. Corzilius, S. Vega, *Handbook of High Field Dynamic Nuclear Polarization* (John Wiley & Sons, 2019).

3. Z. Pang, K. Sheberstov, B. A. Rodin, J. Lumsden, U. Banerjee, D. Abergel, F. Mentink-Vigier, G. Bodenhausen, K. O. Tan, Hypershifted spin spectroscopy with dynamic nuclear polarization at 1.4 K. *Sci. Adv.* **10**, eadr7160 (2024).

4. J. Travesedo, J. O'Sullivan, L. Pallegoix, Z. W. Huang, P. Hogan, P. Goldner, T. Chaneliere, S. Bertaina, D. Estève, P. Abgrall, D. Vion, E. Flurin, P. Bertet, All-microwave spectroscopy and polarization of individual nuclear spins in a solid. *Sci. Adv.* **11**, eadu0581 (2025).

5. W. Mims, Pulsed ENDOR experiments. *Proc. R Soc. London Ser. A Math Phys. Sci.* **283**, 452–457 (1965).

6. A. Meyer, S. Dechert, S. Dey, C. Höbartner, M. Bennati, Measurement of angstrom to nanometer molecular distances with ^{19}F nuclear spins by EPR/ENDOR spectroscopy. *Angew. Chem. Int. Ed. Engl.* **59**, 373–379 (2020).

7. A. Meyer, A. Kehl, C. Cui, F. A. Reichardt, F. Hecker, L.-M. Funk, M. K. Ghosh, K.-T. Pan, H. Urlaub, K. Tittmann, J. Stubbe, M. Bennati, ^{19}F electron-nuclear double resonance reveals interaction between redox-active tyrosines across the α/β interface of E. coli ribonucleotide reductase. *J. Am. Chem. Soc.* **144**, 11270–11282 (2022).

8. J. L. Wort, K. Ackermann, A. Giannoulis, A. J. Stewart, D. G. Norman, B. E. Bode, Sub-micromolar pulse dipolar EPR spectroscopy reveals increasing Cu^{II}-labelling of double-histidine motifs with lower temperature. *Angew. Chem. Int. Ed. Engl.* **131**, 11807–11811 (2019).

9. N. Asanbaeva, A. Sukhanov, A. Diveikina, O. Rogozhnikova, D. Trukhin, V. Tormyshev, A. Chubarov, A. Maryasov, A. Genaev, A. Shernyukov, G. Salnikov, A. Lomzov, D. Pyshnyi, E.

Bagryanskaya, Application of W-band ^{19}F electron nuclear double resonance (ENDOR) spectroscopy to distance measurement using a trityl spin probe and a fluorine label. *Phys. Chem. Chem. Phys.* **24**, 5982–6001 (2022).

10. M. Judd, E. H. Abdelkader, M. Qi, J. R. Harmer, T. Huber, A. Godt, A. Savitsky, G. Otting, N. Cox, Short-range ENDOR distance measurements between Gd(III) and trifluoromethyl labels in proteins. *Phys. Chem. Chem. Phys.* **24**, 25214–25226 (2022).

11. N. Asanbaeva, D. S. Novopashina, O. Y. Rogozhnikova, V. M. Tormyshev, A. Kehl, A. Sukhanov, A. V. Shernyukov, A. Genaev, A. A. Lomzov, M. Bennati, A. Meyer, E. Bagryanskaya, ^{19}F electron nuclear double resonance (ENDOR) spectroscopy for distance measurements using trityl spin labels in DNA duplexes. *Phys. Chem. Chem. Phys.* **25**, 23454–23466 (2023).

12. S. L. Schumann, S. Kotnig, Y. Kutin, M. Drosou, L. M. Stratmann, Y. Streltsova, A. Schnegg, D. A. Pantazis, G. H. Clever, M. Kasanmascheff, Structure and flexibility of copper-modified DNA G-quadruplexes investigated by ^{19}F ENDOR experiments at 34 GHz. *Chemistry* **29**, e202302527 (2023).

13. M. Seal, W. Zhu, A. Dalaloyan, A. Feintuch, A. Bogdanov, V. Frydman, X.-C. Su, A. M. Gronenborn, D. Goldfarb, Gd(III)-^{19}F distance measurements for proteins in cells by electron-nuclear double resonance. *Angew. Chem. Int. Ed. Engl.* **62**, e202218780 (2023).

14. F. J. Tucci, R. J. Jodts, B. M. Hoffman, A. C. Rosenzweig, Product analogue binding identifies the copper active site of particulate methane monooxygenase. *Nat. Catal.* **6**, 1194–1204 (2023).

15. A. Bogdanov, V. Frydman, M. Seal, L. Rapatskiy, A. Schnegg, W. Zhu, M. Iron, A. M. Gronenborn, D. Goldfarb, Extending the range of distances accessible by ^{19}F electron–nuclear double resonance in proteins using high-spin Gd(III) labels. *J. Am. Chem. Soc.* **146**, 6157–6167 (2024).

16. A. Bogdanov, L. Gao, A. Dalaloyan, W. Zhu, M. Seal, X.-C. Su, V. Frydman, Y. Liu, A. M. Gronenborn, D. Goldfarb, Spin labels for ^{19}F ENDOR distance determination: Resolution, sensitivity and distance predictability. *Phys. Chem. Chem. Phys.* **26**, 26921–26932 (2024).

17. M. Gauger, M. Heinz, A.-L. J. Halbritter, L. S. Stelzl, N. Erlenbach, G. Hummer, S. T. Sigurdsson, T. F. Prisner, Structure and internal dynamics of short RNA duplexes determined by a combination of pulsed EPR methods and MD simulations. *Angew. Chem. Int. Ed. Engl.* **136**, e202402498 (2024).

18. L. Remmel, A. Meyer, K. Ackermann, G. Hagelueken, M. Bennati, B. E. Bode, Pulsed EPR methods in the angstrom to nanometre scale shed light on the conformational flexibility of a Fluoride Riboswitch. *Angew. Chem. Int. Ed. Engl.* **63**, e202411241 (2024).

19. D. Rose-Sperling, M. A. Tran, L. M. Lauth, B. Goretzki, U. A. Hellmich, ^{19}F NMR as a versatile tool to study membrane protein structure and dynamics. *Biol. Chem.* **400**, 1277–1288 (2019).

20. H. Welte, T. Zhou, X. Mihajlenko, O. Mayans, M. Kovermann, What does fluorine do to a protein? Thermodynamic, and highly-resolved structural insights into fluorine-labelled variants of the cold shock protein. *Sci. Rep.* **10**, 2640 (2020).

21. A. M. Gronenborn, Small, but powerful and attractive: ^{19}F in biomolecular NMR. *Structure* **30**, 6–14 (2022).

22. M. R. Baker, C. H. Anderson, N. F. Ramsey, Nuclear Magnetic Antishielding of Nuclei in Molecules. Magnetic Moments of ^{19}F, ^{14}N, and ^{15}N. *Phys. Rev.* **133**, A1533 (1964).

23. M. Jaszuński, A. Antušek, P. Garbacz, K. Jackowski, W. Makulski, M. Wilczek, The determination of accurate nuclear magnetic dipole moments and direct measurement of NMR shielding constants. *Prog. Nucl. Magn. Reson. Spectrosc.* **67**, 49–63 (2012).

24. A. Kehl, L. Sielaff, L. Remmel, M. L. Rämisch, M. Bennati, A. Meyer, Frequency and time domain ^{19}F ENDOR spectroscopy: Role of nuclear dipolar couplings to determine distance distributions. *Phys. Chem. Chem. Phys.* **27**, 1415–1425 (2025).

25. A. Kehl, M. Hiller, F. Hecker, I. Tkach, S. Dechert, M. Bennati, A. Meyer, Resolution of chemical shift anisotropy in ^{19}F ENDOR spectroscopy at 263 GHz/9.4 T. *J. Magn. Reson.* **333**, 107091 (2021).

26. P. Höfer, A. Grupp, M. Mehring, High-resolution time-domain electron-nuclear-sublevel spectroscopy by pulsed coherence transfer. *Phys. Rev. A* **33**, 3519–3522 (1986).

27. C. Gemperle, A. Schweiger, Pulsed electron-nuclear double resonance methodology. *Chem. Rev.* **91**, 1481–1505 (1991).

28. P.-P. Zänker, G. Jeschke, D. Goldfarb, Distance measurements between paramagnetic centers and a planar object by matrix Mims electron nuclear double resonance. *J. Chem. Phys.* **122**, 024515 (2005).

29. N. Dayan, Y. Artzi, M. Jbara, D. Cristea, A. Blank, Pulsed electron-nuclear double resonance in the fourier regime. *ChemPhysChem* **24**, e202200624 (2023).

30. M. Pannier, S. Veit, A. Godt, G. Jeschke, H. W. Spiess, Dead-time free measurement of dipole–dipole interactions between electron spins. *J. Magn. Reson.* **213**, 316–325 (2011).

31. H. Thomann, M. Bernardo, Indirect detection of internuclear dipolar couplings in paramagnetic solids. *J. Am. Chem. Soc.* **118**, 5806–5807 (1996).

32. M. Bennati, D. Goldfarb, S. Stoll, "EPR interactions—Hyperfine couplings" in *EPR Spectroscopy: Fundamentals and Methods*, eMagRes Books (John Wiley & Sons, 2017), pp. 271–282.

33. I. Bejenke, R. Zeier, R. Rizzato, S. J. Glaser, M. Bennati, Cross-polarisation ENDOR for spin-1 deuterium nuclei. *Mol. Phys.* **118**, e1763490 (2020).

34. O. Schiemann, C. A. Heubach, D. Abdullin, K. Ackermann, M. Azarkh, E. G. Bagryanskaya, M. Drescher, B. Endeward, J. H. Freed, L. Galazzo, D. Goldfarb, H. Tobias, L. E. Hofer, L. F. Ibáñez, E. J. Hustedt, S. Kucher, I. Kuprov, J. E. Lovett, A. Meyer, S. Ruthstein, S. Saxena, S. Stoll, C. R. Timmel, M. Di Valentin, H. Mchaourab, T. F. Prisner, B. E. Bode, E. Bordignon, M.

Bennati, G. Jeschke, Benchmark test and guidelines for DEER/PELDOR experiments on nitroxide-labeled biomolecules. *J. Am. Chem. Soc.* **143**, 17875–17890 (2021).

35. G. Jeschke, DEER distance measurements on proteins. *Annu. Rev. Phys. Chem.* **63**, 419–446 (2012).

36. J. Pirrwitz, D. Schwarz, Verfahren zur Herstellung von 2,2,6,6-Tetramethyl-4-oxopiperidin. DDR patent DD222017A1 (WP C 07 D/260 901 6) (1984).

37. M. M. Haugland, A. H. El-Sagheer, R. J. Porter, J. Pena, T. Brown, E. A. Anderson, J. E. Lovett, 2'-Alkynylnucleotides: A sequence-and spin label-flexible strategy for EPR spectroscopy in DNA. *J. Am. Chem. Soc.* **138**, 9069–9072 (2016).

38. G. Úr, T. Kalai, K. Hideg, Facile syntheses of 3, 4-disubstituted pyrroline nitroxides and their further synthetic applications. *Tetrahedron Lett.* **57**, 778–780 (2016).

39. L. A. Shundrin, I. A. Kirilyuk, I. A. Grigor'ev, 3-Carboxy-2, 2, 5, 5-tetra ($^{2}H_3$) methyl-[4-^{2}H (^{1}H)]-3-pyrroline-(1-^{15}N)-1-oxyl as a spin probe for *in vivo* L-band electron paramagnetic resonance imaging. *Mendeleev. Commun.* **24**, 298–300 (2014).

40. A. Kehl, "Development of Analysis-and Simulation-Routines for ENDOR Spectroscopy," thesis, Georg-August Universität Göttingen, Göttingen, Germany (2024).

41. M. Bennati, C. Farrar, J. Bryant, S. Inati, V. Weis, G. Gerfen, P. Riggs-Gelasco, J. Stubbe, R. Griffin, Pulsed electron-nuclear double resonance (ENDOR) at 140 GHz. *J. Magn. Reson.* **138**, 232–243 (1999).

42. H. Wiechers, A. Kehl, M. Hiller, B. Eltzner, S. Huckemann, A. Meyer, I. Tkach, M. Bennati, Y. Pokern, Bayesian optimization to estimate hyperfine couplings from 19F ENDOR spectra. *J. Magn. Reson.* **353**, 107491 (2023).

43. A. Feintuch, S. Vega, "Spin dynamics" in *EPR Spectroscopy: Fundamentals and Methods*, eMagRes Books (John Wiley & Sons, 2017), pp. 427–452.

44. M. D. Hanwell, D. E. Curtis, D. C. Lonie, T. Vandermeersch, E. Zurek, G. R. Hutchison, Avogadro: An advanced semantic chemical editor, visualization, and analysis platform. *J. Cheminform.* **4**, 17 (2012).

45. E. Caldeweyher, S. Ehlert, A. Hansen, H. Neugebauer, S. Spicher, C. Bannwarth, S. Grimme, A generally applicable atomic-charge dependent London dispersion correction. *J. Chem. Phys.* **150,** 154122 (2019).

46. S. Stoll, A. Schweiger, EasySpin, a comprehensive software package for spectral simulation and analysis in EPR. *J. Magn. Reson.* **178**, 42–55 (2006).

47. A. Milov, A. Ponomarev, Y. D. Tsvetkov, Electron-electron double resonance in electron spin echo: Model biradical systems and the sensitized photolysis of decalin. *Chem. Phys. Lett.* **110**, 67–72 (1984).

48. F. Weigend, R. Ahlrichs, Balanced basis sets of split valence, triple zeta valence and quadruple zeta valence quality for H to Rn: Design and assessment of accuracy. *Phys. Chem. Chem. Phys.* **7**, 3297–3305 (2005).

49. S. Grimme, J. Antony, S. Ehrlich, H. Krieg, A consistent and accurate ab initio parametrization of density functional dispersion correction (DFT-D) for the 94 elements H-Pu. *J. Chem. Phys.* **132**, 154104 (2010).

50. S. Kossmann, F. Neese, Efficient structure optimization with second-order many-body perturbation theory: The RIJCOSX-MP2 method. *J. Chem. Theory Comput.* **6**, 2325–2338 (2010).
